# Loss of miR-29a/b1 promotes inflammation and fibrosis in acute pancreatitis

**DOI:** 10.1172/jci.insight.149539

**Published:** 2021-10-08

**Authors:** Shatovisha Dey, Lata M. Udari, Primavera RiveraHernandez, Jason J. Kwon, Brandon Willis, Jeffrey J. Easler, Evan L. Fogel, Stephen Pandol, Janaiah Kota

**Affiliations:** 1 Department of Medical and Molecular Genetics, Indiana University (IU) School of Medicine, Indianapolis, Indiana, USA.; 2 Mouse Biology Program, UCD, Davis California, USA.; 3 Department of Medicine, Division of Gastroenterology/Hepatology, IU Health, IU School of Medicine, Indianapolis, Indiana, USA.; 4 The Melvin and Bren Simon Cancer Center, IUSM, Indianapolis, Indiana, USA.; 5 Department of Medicine, Cedar-Sinai Medical Center, Los Angeles, California, USA.

**Keywords:** Gastroenterology, Genetics, Extracellular matrix, Genetic variation, Molecular pathology

## Abstract

MicroRNA-29 (miR-29) is a critical regulator of fibroinflammatory processes in human diseases. In this study, we found a decrease in miR-29a in experimental and human chronic pancreatitis, leading us to investigate the regulatory role of the miR-29a/b1 cluster in acute pancreatitis (AP) utilizing a conditional miR-29a/b1–KO mouse model. miR-29a/b1-sufficient (WT) and -deficient (KO) mice were administered supramaximal caerulein to induce AP and characterized at different time points, utilizing an array of IHC and biochemical analyses for AP parameters. In caerulein-induced WT mice, miR-29a remained dramatically downregulated at injury. Despite high-inflammatory milieu, fibrosis, and parenchymal disarray in the WT mice during early AP, the pancreata fully restored during recovery. miR-29a/b1–KO mice showed significantly greater inflammation, lymphocyte infiltration, macrophage polarization, and ECM deposition, continuing until late recovery with persistent parenchymal disorganization. The increased pancreatic fibrosis was accompanied by enhanced TGFβ1 coupled with persistent αSMA^+^ PSC activation. Additionally, these mice exhibited higher circulating IL-6 and inflammation in lung parenchyma. Together, this collection of studies indicates that depletion of miR-29a/b1 cluster impacts the fibroinflammatory mechanisms of AP, resulting in (a) aggravated pathogenesis and (b) delayed recovery from the disease, suggesting a protective role of the molecule against AP.

## Introduction

Acute pancreatitis (AP), characterized by complex pathophysiological processes, oftentimes results in systemic complications and multiorgan failure (1). AP is also shown as an early presenting symptom for pancreatic ductal adenocarcinoma (PDAC) (2). Studies using genetically engineered mice have demonstrated that brief episodes of AP can induce speedy progression of pancreatic intraepithelial neoplasia and PDAC (3, 4). Although considerable advances have been made to delineate the pathophysiology of AP, effective drugs are not available to date, primarily because of the dearth of precise understanding of the molecular mechanisms of the disease. Thus, elucidating the key pathological mechanisms of AP is essential for clinical management and reducing the risk for PDAC development.

MicroRNAs (miRNAs) are a class of highly conserved noncoding RNAs that regulate expression of multiple genes through translational repression or mRNA degradation. Aberrant miRNA expression results in alternation of essential physiological processes in numerous pathological conditions, including AP (5, 6). The miR-29 family consists of 3 members — 29a, 29b, and 29c — encoded by 2 polycistronic miRNA clusters, miR-29a/b1 and miR-29b2/c. Among the 3, miR-29a is the most abundantly expressed member in the pancreas, pancreatic epithelium, and pancreatic stellate cells (PSCs) (7, 8) and originates solely from the miR-29a/b1 cluster. In recent years, miR-29a is shown to function as an antifibrotic and antiinflammatory molecule in different organs (9, 10), particularly in diseases such as PDAC (7, 8, 11). As pancreatitis is a major risk factor for PDAC development, and miR-29a is critical to fibroinflammatory mechanisms and the primary member in the pancreas, we asked whether depletion of miR-29a has a role in pancreatitis, with specific focus on characterizing the function of miR-29a/b1 cluster in AP pathogenesis.

miR-29a was downregulated in both murine and human pancreatitis. To gain deeper insights, we generated a potentially novel, pancreas-specific, conditional miR-29a/b1–KO mouse model and induced AP with i.p. caerulein injections. AP induced by caerulein hyperstimulation has been well characterized and mimics clinical forms of the disease (12). Here, we report the pathological and molecular changes in miR-29a/b1–KO mice in comparison with miR-29a/b1 sufficient (WT) mice during AP utilizing a 10-day time-course experiment. We found that AP causes miR-29a loss and that the loss of the miR-29a/b1 cluster during AP accelerates pancreatic injury and delays recovery, with increased inflammatory infiltration and macrophage polarization during the course of the disease. We further identified that loss of miR-29a/b1 results in TGFβ1-mediated PSC activation congruent with enhanced and prolonged ECM accumulation during AP progression. Our findings delineate a potentially novel protective role of miR-29 in fibroinflammatory regulation of AP, which may form the basis for the development of new targeted therapeutic strategies for the disease.

## Results

### miR-29a is transiently downregulated during the early phase of pancreatitis.

**Figure 1:**
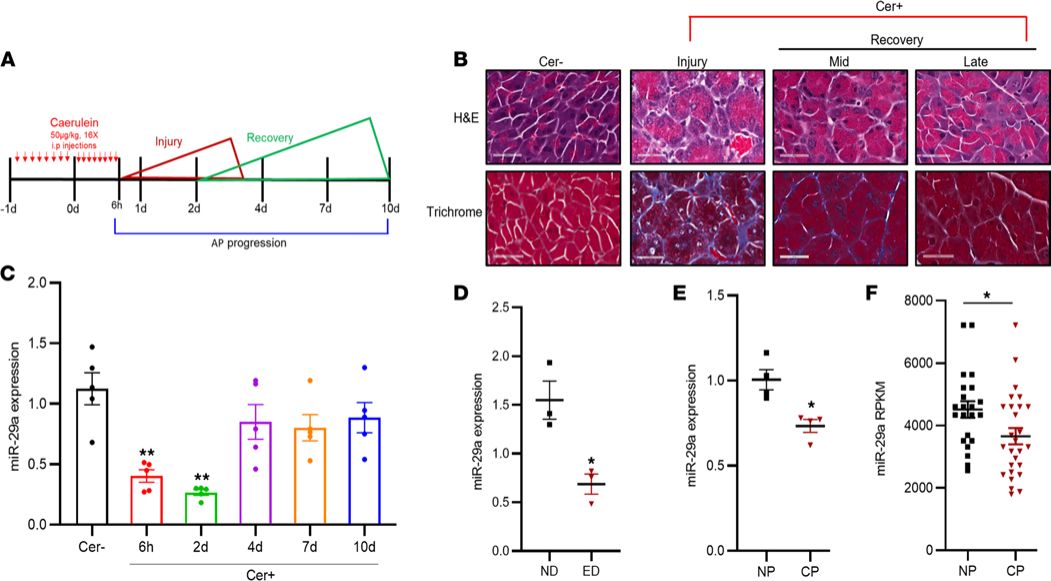
Pathobiological role of miR-29a/b1 in AP. (**A**) Schematic representation of caerulein hypersimulation for induction of AP and the disease progression through acute and recovery phases. (**B**) Representative IHC images for H&E and Masson’s trichrome staining depicting pancreatic tissue sections of saline-treated (Cer–) and caerulein dosed (Cer+) WT mice at injury and recovery phases of AP. Total original magnification, ×200. Scale bar: 200 μm. Images represent *n* = 5/group/time point. (**C**) qPCR analysis of miR-29a expression in pancreata of WT mice during the course of AP (*n* = 5mice/group/time point). (**D**) qPCR analysis of miR-29a expression in pancreata of WT mice fed with normal diet (ND) or ethanol-rich diet (ED) (*n* = 3/group). (**E**) qPCR quantification of miR-29a expression in tissue samples from patients with chronic pancreatitis (CP) compared with normal adjacent pancreas (NP); *n* = 4. (**F**) miR-29a expression in the pancreas of patients with chronic pancreatitis (CP; *n* = 27) compared with normal donor pancreas (NP; *n* = 22) (https://www.ncbi.nlm.nih.gov/geo/query/acc.cgi?acc=GSE24279). *P < 0.05, 1-way ANOVA with Dunnett’s post hoc test (**C**), 2-tailed Student’s *t* test (**D**–**F**).

To investigate the biological significance of miR-29 in pancreatitis, miR-29a expression and histological analyses were performed in a 10-day time-course experiment representing acute, early, and late recovery phases of AP (Figure 1, A and B). Compared with PBS-treated WT mice (controls), miR-29a expression in caerulein-treated WT mice was robustly reduced during pancreatic injury (60% at 6 hours and 70% at 2 days of control levels) (Figure 1C). Subsequently, as the pathology resolved, miR-29a expression was restored to reach close to baseline during the recovery phases (4, 7, and 10 days). Next, we sought to find if the aberrant expression of miR-29a is relevant for pancreatitis originating from other etiologies — for example, natural environmental stressors. To this end, we examined the levels of miR-29a in the pancreata of mice fed with an alcohol-rich (ethanol-rich) diet to induce pancreatitis. Similar to caerulein-induced mice, marked reduction in miR-29a expression was also found in the pancreata of these mice (Figure 1D). A significant downregulation of miR-29a was also observed in human chronic pancreatitis tissue samples compared with normal adjacent pancreas tissues (Figure 1E and Supplemental Tables 1 and 2; supplemental material available online with this article; https://doi.org/10.1172/jci.insight.149539DS1) or in comparison with healthy donor pancreata (13) (Figure 1F), indicating the global relevance of the molecule in pancreatitis.

### Generation of pancreas-specific miR-29a/b1–KO mice.

**Figure 2:**
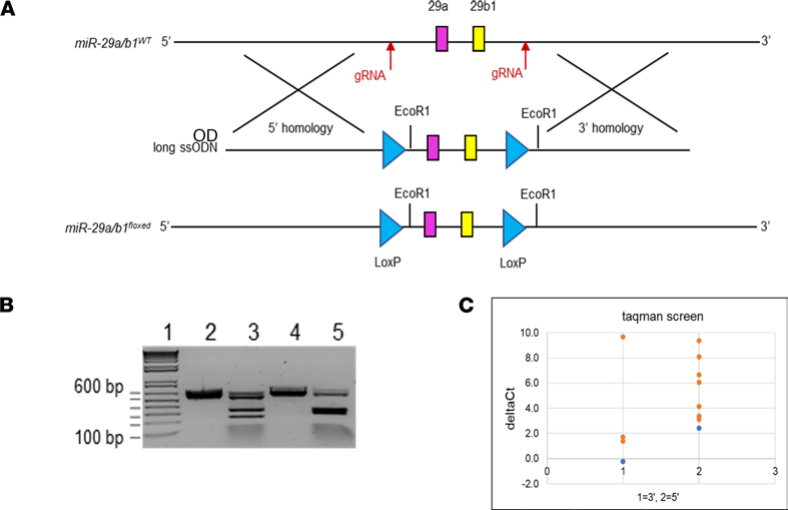
Generation of miR-29a/b1–floxed allele for development of miR-29a/b1–KO murine model. (**A**) Schematic representation for the generation of miR-29a/b1–floxed allele using CRISPR/Cas9 technology. (**B**) PCR analysis for validation of cleaved fragment consisting of guide RNA generated by cleavage assay. Lane 1: 1 kb ladder with 100 bp fragment at the bottom (labeled), with 200, 300, 400, 500, and 600 bp (labeled) at the top respectively. Lane 2: miR-29b (5′) Control, 563 bp. Lane 3: miR-29b (5′) crRNA, 333/230 bp (80% cleavage). Lane 4: miR-29b (3′) Control, 625 bp. Lane 5: miR-29a (3′) crRNA, 336/289 bp (80% cleavage). (**C**) TaqMan assays were designed to screen for allelic fragments consisting of both 5′ and 3′ LoxP sites, utilizing a unique LoxP dual-labeled probe for each site. ΔCt for each LoxP site was measured by quantification of the (Fam-labeled) target LoxP Ct value relative to that of an endogenous housekeeping gene Tcrd (VIC labeled) in each sample. Presence of the target locus was screened based on the ΔCt value, where the value closest to zero (least difference between the genomic copies of the housekeeping gene and the target site) represented significant genomic copy number for the site. Each blue dot for the 3′ and 5′ LoxP sites represents the positive controls used in the assay. Orange dots for each site depict some of the screened animals, with the 2 orange dots exactly above the positive control (blue dots) for each 3′ and 5′ sites that were double-positive for the 2 LoxP sites.

**Figure 3:**
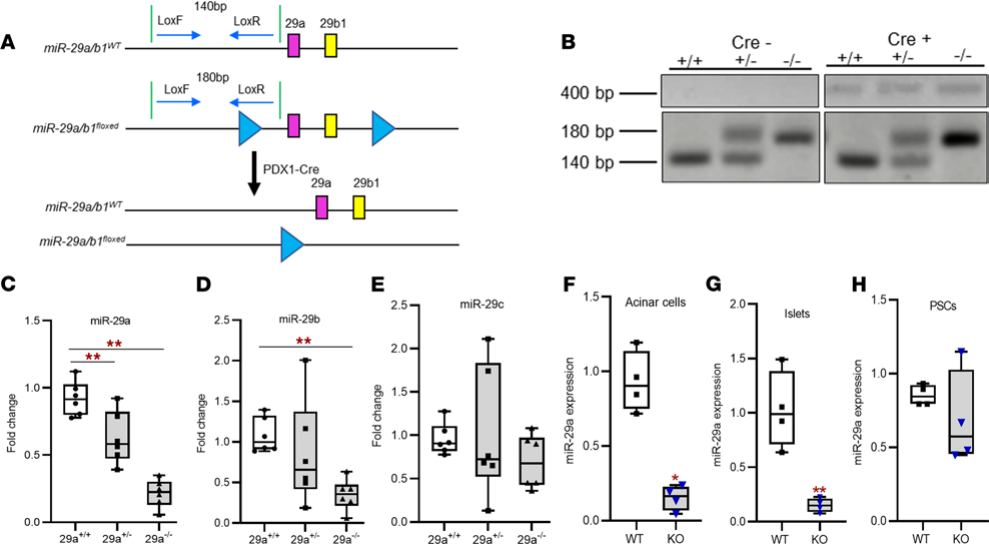
Validation of transgenic miR-29a/b1–KO mouse model. (**A**) Schematic for the generation of conditional pancreas-specific miR-29a–KO transgenic mice. Representation of miR-29a/b1 WT allele (top), miR-29a/b1–floxed allele with 2 LoxP sites (blue triangles) flaking miR-29a/b1 cluster (middle) and miR-29a/b1–floxed allele after Cre recombination (bottom). miR-29a/b1–floxed mice were crossed with a mouse strain that express Cre recombinase under pancreas-specific promoter (Pdx1-Cre) to generate miR-29a/b1–KO mice. (**B**) Genotypes of the miR-29a/b1–KO mice were confirmed from tail snips utilizing a PCR strategy to generate fragments with specific sizes for WT allele (*miR-29ab1^+/+^*; 140 bp), heterozygous allele (*miR-29ab1^+/–^*; 140 and 180 bps), and homozygous miR-29a/b1–KO allele (*miR-29ab1^–/–^*; 180 bp). Cre^+^ was determined by the presence or absence of a 410 bp fragment. (**C**–**E**) Expression of miR-29a, -b, and -c respectively in WT (miR-29ab1^+/+^, Cre-; *n* = 6), miR-29a/b1 Het (miR-29ab1^+/–^, Cre+; *n* = 6), and miR-29a/b1–KO (miR-29ab1^–/–^, Cre+; *n* = 6) mice confirmed by qPCR analysis of total RNA obtained from the mouse pancreata (*n* = 6). (**F**–**H**) miR-29a expression in WT and miR-29a/b1–KO mouse acinar cells (*n* = 4) (**F**), islets (*n* = 4) (**G**), and PSCs (*n* = 4) (**H**) isolated from the pancreas and measured by qPCR analysis (*n* = 4). Graphs represent mean ± SEM; *P < 0.05; **P < 0.01, 1-way ANOVA with Tukey’s post hoc test (**C**–**E**), 2-tailed Student’s *t* test (**F**–**H**).

With a consistent loss of miR-29a in both human and murine pancreatitis, we aimed to characterize the function of the miR-29a/b1 cluster in a physiologically relevant murine model. To this end, utilizing CRISPR/Cas9 technology, we developed a conditional miR-29a/b1–floxed mouse model as described in Methods and Figure 2. The miR-29a/b1 cluster encodes for both mature miR-29a and miR-29b. Mature miR-29b also originates from another cluster (miR-29b2/c), but mature miR-29a originates exclusively from the miR-29a/b1 cluster. miR-29a/b1–floxed mice were crossed with a mouse strain expressing Cre recombinase under the pancreas-specific promoter *Pdx1* (Pdx1-Cre) to allow endogenous depletion of miR-29a in the pancreas (Figure 3A). Pancreas-specific miR-29a/b1–KO mice were identified based on genomic analysis for Cre and LoxP alleles from mouse tail snips (Figure 3B), followed by quantitative PCR (qPCR) analysis for the miR-29 family members in the mouse pancreas postmortem (Figure 3, C–E). The KO mice exhibited ~80% depletion of endogenous miR-29a in the pancreas (Figure 3C). miR-29b, which is also partially encoded in the targeted cluster, was depleted by ~60% in this KO model (Figure 3D).

The transcription factor (TF) *Pdx1* plays essential roles in pancreatic tissue development and differentiation of pancreatic cell lineages (14). In adult mice, *Pdx1* is expressed only in specific cell types, such as acinar cells, β cells of the islets, and ductal cells (15–17). To examine the specificity of Pdx1-Cre–mediated miR-29a depletion in our murine model, we measured miR-29a levels in acinar cells, islets, and PSCs in the mouse pancreata. Expectedly, miR-29a was downregulated in acinar cells and islets, with no change in PSCs of miR-29a–KO mice, validating the aptness of the murine model described in the current work (Figure 3, F–H).

### The miR-29a/b1 cluster is dispensable in normal adult pancreas.

**Figure 4:**
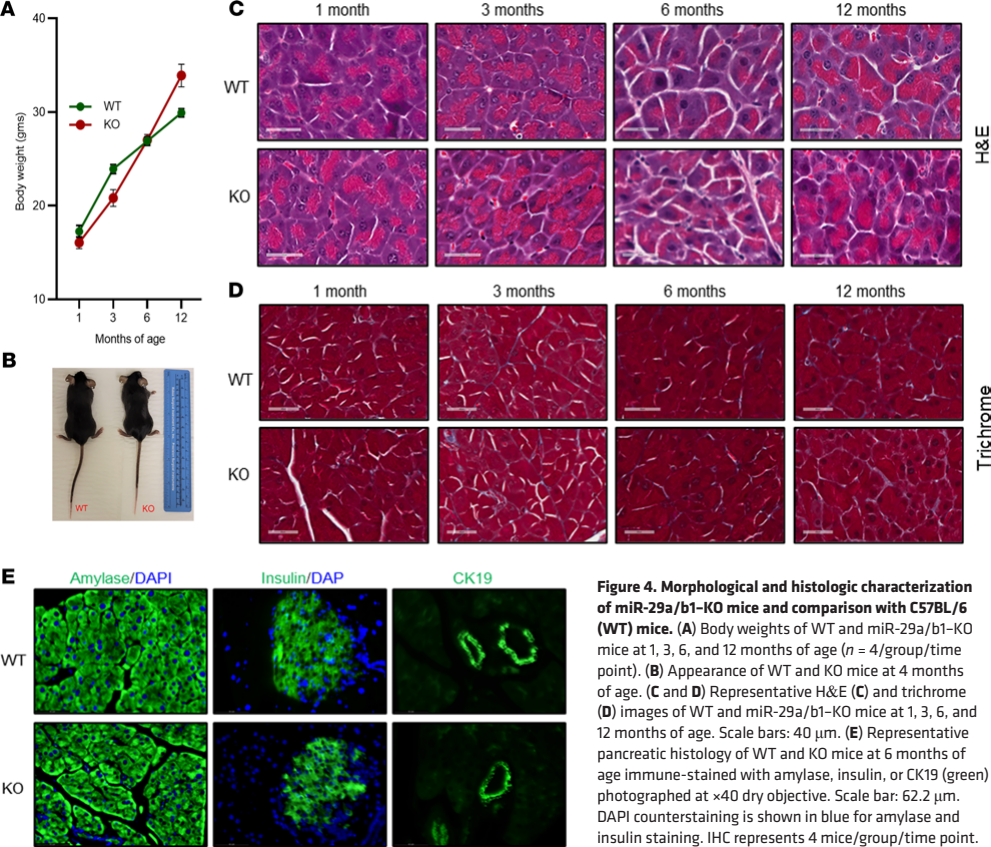
Morphological and histologic characterization of miR-29a/b1–KO mice and comparison with C57BL/6 (WT) mice. (**A**) Body weights of WT and miR-29a/b1–KO mice at 1, 3, 6, and 12 months of age (*n* = 4/group/time point). (**B**) Appearance of WT and KO mice at 4 months of age. (**C** and **D**) Representative H&E (**C**) and trichrome (**D**) images of WT and miR-29a/b1–KO mice at 1, 3, 6, and 12 months of age. Scale bars: 40 μm. (**E**) Representative pancreatic histology of WT and KO mice at 6 months of age immune-stained with amylase, insulin, or CK19 (green) photographed at ×40 dry objective. Scale bar: 62.2 μm. DAPI counterstaining is shown in blue for amylase and insulin staining. IHC represents 4 mice/group/time point.

To determine the biological significance of miR-29a/b1 cluster in normal pancreas, we characterized miR-29a/b1–KO mice in a time-course experiment at 1, 3, 6, and 12 months of age. Pancreas-specific miR-29a/b1–KO mice were viable and fertile, with normal body weight (Figure 4A) with similar size, behavior, and gross morphology as their WT littermates (Figure 4B). H&E staining of the pancreas of the KO mice revealed normal morphology of the pancreatic cells (Figure 4C) without any aberrant fibrotic patterns (Figure 4D). The structure of acinar cells, islets, and the ductal system were normal in these mice and each was verified by immunostaining pancreatic sections with antibodies against amylase, insulin, and CK19, respectively (Figure 4E and Supplemental Figure 1). Though the distribution of the different cell types was normal in both the groups, the islet sizes generally appeared slightly larger in the younger KO mice at 1 and 3 months of age. In both the groups, older mice appeared to have larger, less abundant islets.

### Loss of miR-29a/b1 exacerbates pancreatic injury and impairs pancreatic regeneration in AP mice.

Our initial observations indicated that pancreatic depletion of the miR-29a/b1 cluster alone does not result in deleterious outcomes. However, experimental evidence implicates that miRNAs play crucial roles in response to stress, environmental insults, or changes in microenvironment (18, 19). Accordingly, their misregulation may exacerbate disease pathology (18, 19). Supporting this notion, we previously observed significant downregulation of miR-29a in PDAC (8, 20, 21). Therefore, we sought to examine the biological significance of the molecule in pancreatitis in a physiologically relevant context. Therefore, miR-29a/b1–KO and WT mice were challenged with acute doses of caerulein to induce AP, and pathophysiological changes were analyzed using established AP time-course experiment (Figure 1A). WT and KO mice were challenged with caerulein or PBS and assessed for AP severity during its pathogenesis.

**Figure 5:**
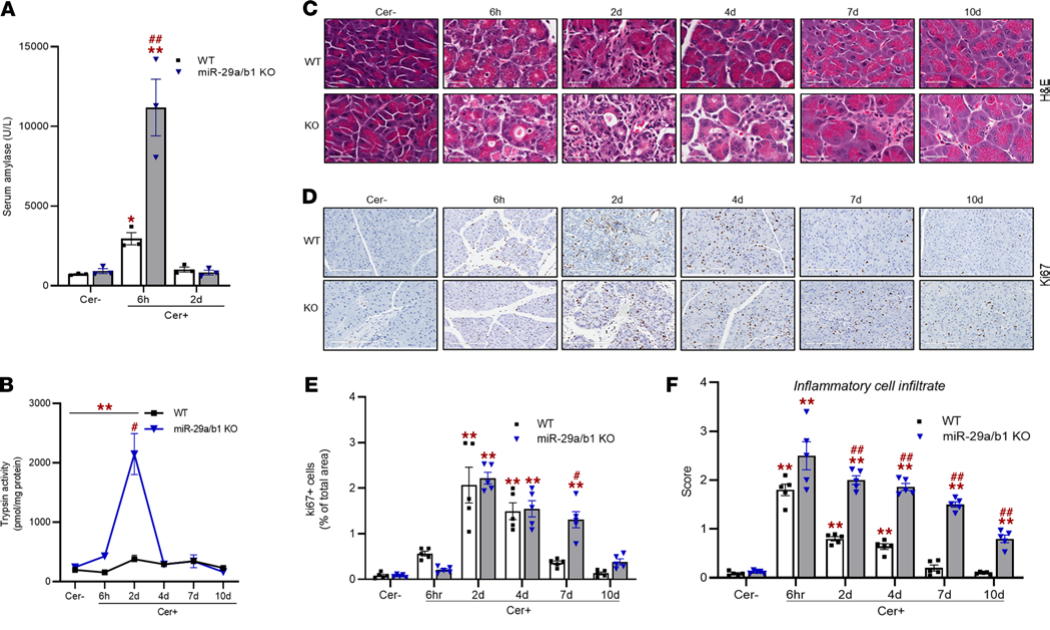
Depletion of miR-29a/b1 aggravates pancreatic injury in AP mice. (**A**) Serum amylase activity in WT and miR-29a/b1–KO mice at 6 hours and 2 days after i.p. injections (*n* = 3). (**B**) Pancreatic trypsin activity in WT and KO mice during the course of AP progression (*n* = 3). (**C**) Representative H&E images of pancreatic sections of WT and KO mice treated with saline (Cer–), or at 6 hours, 2 days, 4 days, 7 days, and 10 days after caerulein administration. Scale bars: 40 μm. (**D** and **E**) Representative staining of Ki67^+^ cells in pancreatic tissue sections of saline- (Cer–) and caerulein-dosed (Cer+) WT and KO mice through the course of AP progression and corresponding quantification per 5 high-powered fields (*n* = 5 mice/group/time point). (**F**) Quantification of inflammatory immune cell infiltration from H&E-stained pancreatic sections of saline (Cer–) and caerulein (Cer+) dosed WT and miR-29a/b1–KO mice through the course of AP progression. Graphs represent mean ± SEM. Asterisk denotes difference with saline-treated WT mice; # symbol denotes difference between caerulein dosed WT and miR-29a–KO mice at a given time point; ^#^*P* < 0.05, **P < 0.01, and ^##^*P* < 0.01, 2-way ANOVA with Bonferroni post hoc test.

Phenotypically, both WT and KO mice exhibited similar behavior with no difference in gross body weights through the course of AP. Also, no mortality was observed in either group. To test the potential effect of miR-29a/b1 loss on AP severity, we measured the parameters of AP in both WT and KO groups at different time points in response to caerulein or PBS. Quantification of the serum amylase levels showed no difference in the saline-treated (control) WT and KO mice (Figure 5A). However, during the acute phase (6 hours after final caerulein injection), amylase levels were markedly increased in both the WT and KO groups, with a greater increase in KO mice compared with the WT. By 2 days, the serum amylase levels reached close to basal values in both groups (Figure 5A). A significant rise in trypsin activity was observed in the KO mice at the 2-day time point, but no significant alteration in trypsin activity was found in the mice groups at any other time points (Figure 5B).

Histological examination of the pancreata of WT mice revealed extensive disorganization of the parenchymal structure, increased interstitial expansion (indication of edema), pseudo tubular complexes, pancreatic acinar cell damage, and vacuolization during the acute phase (6 hours) of AP. These phenotypes in the WT mice subsequently subsided during the early recovery phases (2 and 4 days) with gradual parenchymal reorganization, restoration of acinar cells, and clearance of cellular debris. The tissue became indistinguishable from the saline-treated control group by day 7 (Figure 5C). Compared with WT, caerulein-dosed miR-29a/b1–KO mice showed remarkably increased parenchymal disarray and interstitial expansion during the acute phase (6 hours), with greater histological damage, acinar cell vacuolation, degradation, and necrotic areas. Furthermore, pancreata of these mice remained severely impaired during early recovery (2 days). Although in miR-29a/b1–KO mice, pancreatic restoration was observed starting from 4 days, the phenotype did not reverse back to completely normal during recovery (Figure 5C).

Congruently, compared with the WT mice, the miR-29a/b1–KO mice exhibited a slightly diminished number of cells positive for the proliferative marker Ki67 during acute pancreatic injury (6 hours), but the number of positive cells in WT and KO mice were even during early recovery phases (2 and 4 days). At 7 days, Ki67^+^ cells in WT mice were much diminished, while they were persistent in the KO group. These results suggest a delay in recovery in the KO mice (Figure 5, D and E).

The current study primarily characterizes the role of miR-29a/b1 in the caerulein-induced AP model system; however, we examined the association of this miRNA cluster in another model of AP generated by induction of L-arginine (22). Similar to the caerulein model, there was reduction of miR-29a expression in WT mice dosed with L-arginine compared with saline-treated control group (Supplemental Figure 2A). Histological comparison of the pancreata of WT and KO mice dosed with L-arginine 72 hours after final injection revealed more disorganized pancreata in the KO group characterized by extensive damage in pancreatic acini and cellular degradation (Supplemental Figure 2, B and C). These observations further suggest a global relevance of the miR-29a/b1 in AP pathogenesis.

### Loss of miR-29a/b1 results in enhanced pancreatic inflammatory milieu in AP mice.

To examine whether loss of miR-29a/b1 impacts inflammation, we measured the extent of myeloid cell infiltration from H&E-stained pancreata of the 2 mice groups dosed with caerulein. During the acute phase (6 hours), maximal infiltration of the inflammatory cells was observed in both the WT and miR-29a/b1–KO groups. Although infiltration gradually diminished in WT mice during recovery, reaching basal levels at 10 days, in the KO group, the cell numbers remained significantly higher (Figure 5F).

**Figure 6:**
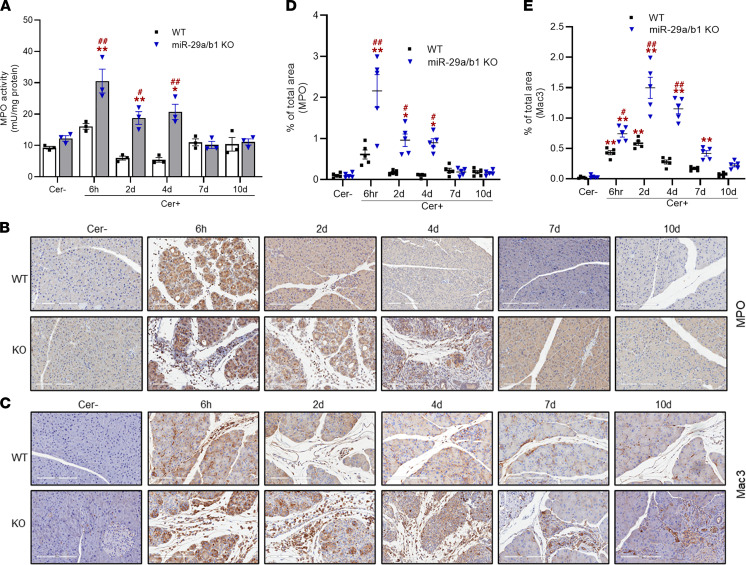
Loss of miR-29a/b1 promotes enhanced and prolonged infiltration of immune cells in the pancreas of AP mice. (**A**) MPO activity in the pancreatic homogenates expressed as milli units (mU) of MPO activity per mg tissue protein (*n* = 3/group/time point). (**B** and **C**) Representative IHC staining of MPO and Mac3 in pancreatic tissue sections of saline- (Cer–) and caerulein-dosed (Cer+) WT and KO mice through the course of AP progression. Scale bars: 200 μm. (**D** and **E**) Corresponding area score for MPO and MAC3 staining from IHC analysis quantified per 5 high-powered fields (*n* = 5 mice/group/time point). Graphs represent mean ± SEM. Asterisk denotes difference with saline-treated WT mice (Controls); # symbol denotes difference between caerulein dosed WT and miR-29a–KO mice at a given time point; *P < 0.05, ^#^*P* < 0.05, **P < 0.01, and ^##^*P* < 0.01, 2-way ANOVA with Bonferroni post hoc test.

In both groups during the acute phase, neutrophils were the majority among all the inflammatory cells. Pancreatic myeloperoxidase (MPO) activity was significantly higher in the KO mice during injury (Figure 6A). This was further confirmed by IHC analysis for MPO in the pancreatic sections (Figure 6, B and D). In WT mice, the infiltration of the neutrophils diminished considerably during early recovery (2 and 4 days) and reached basal levels at later phases (7 and 10 days). However, in the KO group, though reduced, the neutrophil infiltration remained significantly high for up to 4 days after caerulein injection (Figure 6, A, B, and D).

**Figure 7:**
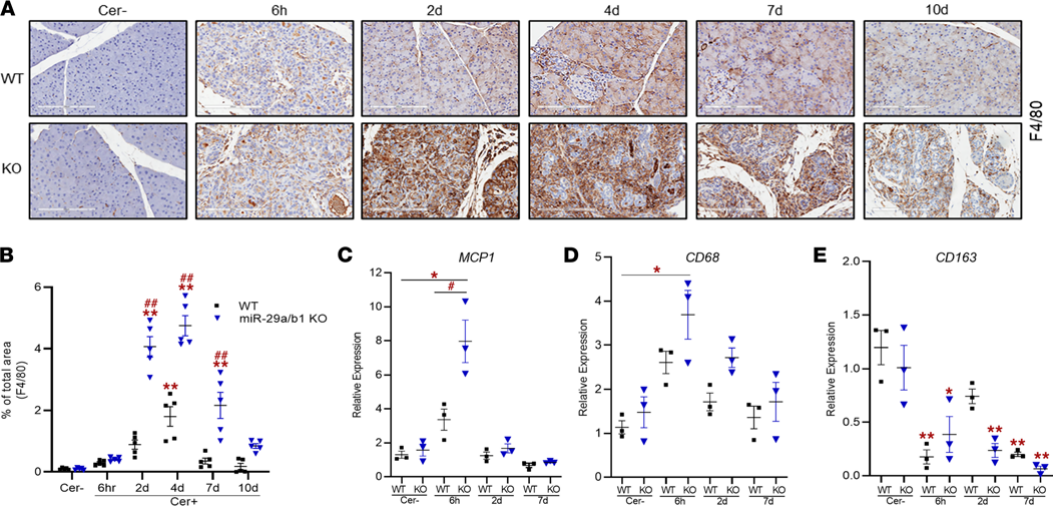
miR-29a/b1 deficiency leads to altered expressions of MCP1 and macrophage populations in AP mice. (**A**) Representative F4/80 staining of saline-treated (Cer–) or caerulein-dosed WT and miR-29a/b1–KO mice through AP time course. Scale bars: 200 μm (*n* = 5 animals/group/time point). (**B**) Corresponding positively stained macrophages from IHC analysis quantified per 5 high-powered fields. (**C**–**E**) Relative expressions of *MCP1*, *CD68*, and *CD163* as assessed by qPCR analysis of pancreatic tissues of saline-treated (Cer–) or caerulein-dosed WT and miR-29a/b1–KO mice at acute (6 hours), early recovery (2 days), and late recovery (7 days) phases of AP (*n* = 3 mice/group/time point). Graphs represent mean ± SEM. Asterisk denotes difference with saline-treated WT mice; # symbol denotes difference between caerulein dosed WT and miR-29a–KO mice at a given time point; *P < 0.05, ^#^*P* < 0.05, **P < 0.01, and ^##^*P* < 0.01, 2-way ANOVA with Bonferroni post hoc test.

In both the mouse groups, pancreatic macrophage infiltration was measured by IHC analysis of 2 markers, *MAC3* and *F4/80* (Figure 6, C and E, and Figure 7, A and B). At 2 and 4 days after caerulein injections, macrophage infiltration was predominant among all the measured leukocytes, with significantly higher numbers in the KO group. Macrophage density eventually declined in the WT mice during recovery but remained persistent for up to 7 days in the KO group.

### miR-29a/b1 deficiency induces macrophage polarization during AP.

Pancreatic acinar injury triggers activation of proinflammatory cytokines, which are critical components of the complex cascade of immunological events in AP. In disease conditions, such as pancreatitis, these inflammatory reactions lead to the development of fibrosis. Monocyte chemoattractant protein 1 (*MCP1*) is an inflammatory cytokine that regulates local and systemic inflammation and fibrosis during pancreatitis (23). Measuring the expression pattern revealed a transient upregulation of the cytokine in the KO mice (6-fold) during the acute (6 hours) phase compared with the WT group. Though there was a 2.5-fold increase in the WT mice at 6 hours, the expression did not reach statistical significance (Figure 7C). The *MCP1* expression returned to basal levels in both groups during the recovery phases.

In addition to proinflammatory cytokines, macrophages play crucial role during the progression of AP (24). In response to environmental stimuli, macrophages can change their plasticity and physiology to exhibit distinct macrophage phenotypes (25). Classically activated macrophages (M1 macrophages) are induced by proinflammatory cytokines during early inflammation, while alternatively activated macrophages (M2 macrophages) are activated at later stages of immune response. Therefore, we next set out to determine if loss of miR-29a/b1 affects activation of the macrophage classes during AP progression. In the KO mice, there was a stark upregulation of CD68^+^ M1 macrophage during the acute phase (6 hours), while the expression was reduced at 2 days (Figure 7D). No difference in M1 macrophage expression was observed at later time points. Interestingly, CD163^+^ M2 macrophage remained significantly reduced in WT mice during acute (6 hours) and late recovery (7 days) phases (Figure 7E). However, in KO mice, the reduction in *CD163* expression was consistent through injury (6 hours and 2 days) and recovery (7 days) (Figure 7E).

### miR-29a/b1 depletion enhances ECM deposition and fibrosis in AP.

**Figure 8:**
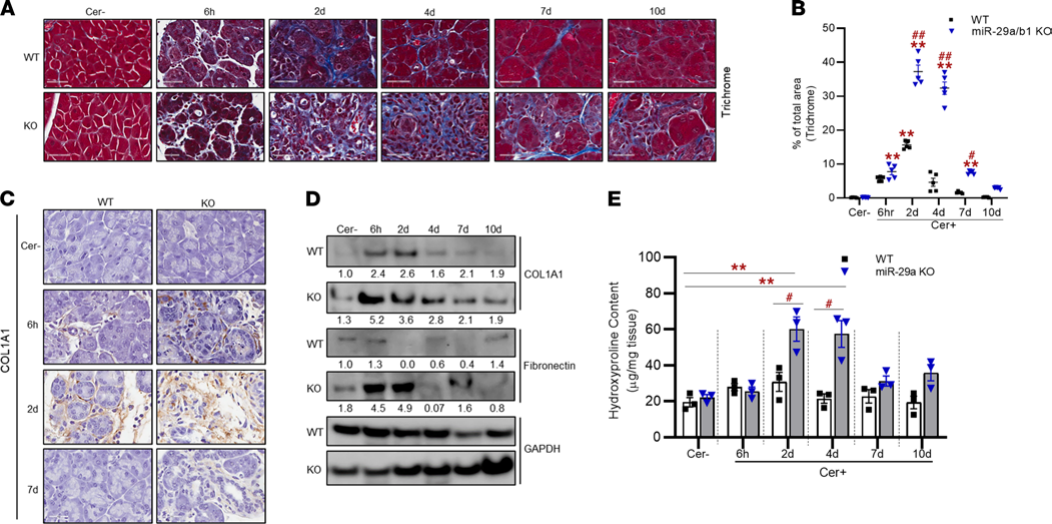
Loss of miR-29a/b1 promotes fibrosis in AP mice. (**A**) Representative Masson’s trichrome staining in pancreatic sections of WT and miR-29a/b1–KO mice dosed with saline (Cer–) or treated with caerulein at 6 hours, 2 days, 4 days, 7 days, and 10 days after i.p. injections (*n* = 5/group/time point). Scale bars: 40 μm. (**B**) Collagen^+^ area (blue) was quantified from trichrome stained sections in 5 high-powered fields (*n* = 5 mice/group/time point). (**C**) Collagen content in the tissue section was confirmed by IHC staining of COL1A1. Scale bars: 40 μm (*n* = 3 animals/group/time point). (**D**) Western blot analysis from pancreatic homogenate for COL1A1 and fibronectin. GAPDH was used as the loading control. Western blot and IHC images are representative of 3 mice/group/time point. (**E**) Total collagen was quantified by measuring hydroxyproline content in the pancreatic tissue sections (*n* = 3 mice/group/time point). Graphs represent mean ± SEM. Asterisk denotes difference with saline-treated WT mice; # symbol denotes difference between caerulein dosed WT and miR-29a–KO mice at a given time point; *P < 0.05, **P < 0.01, and ^##^*P* < 0.01, 2-way ANOVA with Bonferroni post hoc test.

Pancreatic fibrosis is salient to pancreatic injury in AP. To test if miR-29 plays a role in fibrotic processes of AP, we first performed histological analysis of the pancreatic sections of caerulein- and saline-treated mice by staining with Masson’s trichrome to measure fibrosis. None of the saline-treated mice showed signs of fibrosis. In the WT mice, transient collagen deposition was observed in the acinar luminal spaces during injury, with maximal deposition at 2 days, which then gradually subsided starting at 4 days after caerulein injection (Figure 8, A and B). No collagen was observed at later recovery. In miR-29a/b1–KO mice, collagen deposition surged at 2 days, with intense Masson’s trichrome staining in and around acinar lumen. In contrary to the WT group, substantial collagen was present at 4 days in the KO group (Figure 8, A and B), and it continued to accumulate until recovery. These observations suggest that loss of miR-29a/b1 impairs pancreatic recovery from fibrotic processes in AP.

The findings were further validated with histological staining of the pancreas sections and Western blot analysis of pancreatic lysates with the collagen protein collagen type I α 1 (COL1A1) (Figure 8, C and D). In IHC analysis, COL1A1 appeared as fibrillar structures surrounding the acinar cells and islets. COL1A1 deposition surged during injury in both the groups, with higher expression in the KO mice at 6-hour and 2-day time points. COL1A1 went back to normal or close to normal at recovery in the WT mice, while it remained slightly higher in the KO mice. Measurement of fibronectin revealed highly elevated levels of this ECM protein in the miR-29a/b1–KO mice during AP injury (6 hours and 2 days) (Figure 8D).

We next measured hydroxyproline content in the mouse pancreata, which revealed a significant increase in the hydroxyproline content at 2- and 4-day time points in the KO group (Figure 8E). Collectively, these data suggest that loss of miR-29a results in a marked increase in pancreatic ECM protein deposition and fibrotic mechanisms to delay recovery of the injured pancreas during AP.

### miR-29a/b1–KO promotes TGFβ1-mediated activation of PSCs in AP.

Activated PSCs are the major cell type responsible for ECM deposition and pancreatic fibrogenesis (21). In response to pancreatic injury, PSCs are activated by growth factors and cytokines via autocrine and paracrine mechanisms (26). *TGFβ1*, a key PSC activating factor (27), is highly expressed and associated with fibrotic mechanisms of pancreatitis, as well as PDAC stromal reaction (28, 29). PSCs are shown to regulate fibroinflammatory processes of pancreatitis (30). We next aimed to assess the PSC–miR-29 mechanistic axis in the context of AP.

**Figure 9:**
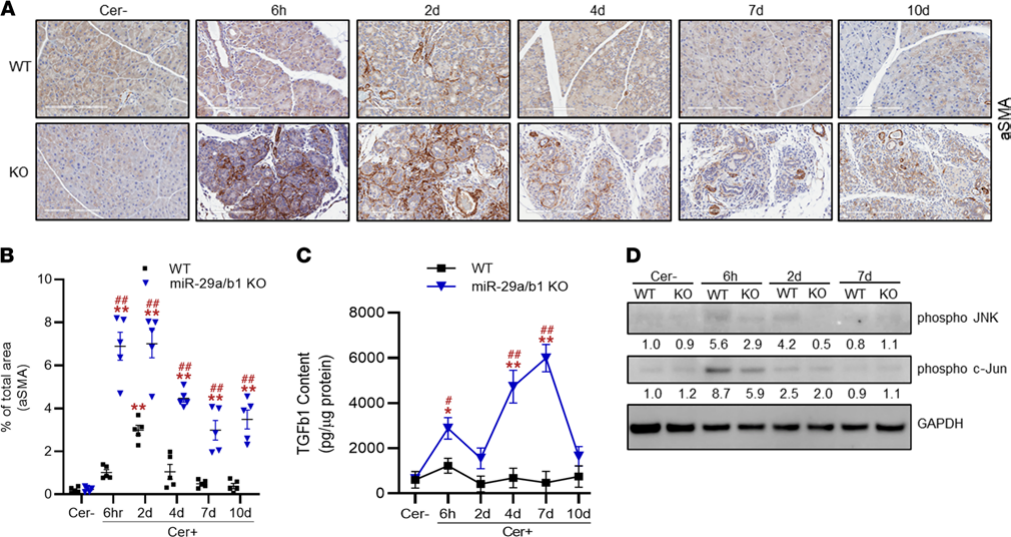
Loss of miR-29a induces enhanced activation of pancreatic stellate cells (PSCs) in AP mice. (**A** and **B**) Representative IHC images for αSMA staining in pancreatic sections of WT and miR-29a/b1–KO mice treated with saline (Cer–) or at 6 hours, 2 days, 4 days, 7 days, and 10 days after caerulein injections. Scale bars: 200 μm. αSMA^+^ area was quantified per 5 high-powered fields. IHC images represent *n* = 5 animals/group/time point. (**C**) TGFβ1 levels were measured in pancreatic homogenates by ELISA for WT and miR-29a/b1–KO mice treated with saline (Cer–) or caerulein at indicated time points (*n* = 3 animals/group/time point). (**D**) Western blot analysis from pancreatic homogenate for phospho–p46 JNK, and phospho–c-Jun. GAPDH was used as the loading control. Western blot images are representative of 3 mice/group/time point. Results in graph represent mean ± SEM. Asterisk denotes difference with saline-treated WT mice; # symbol denotes difference between caerulein dosed WT and miR-29a–KO mice at a given time point; *P < 0.05, ^#^*P* < 0.05, **P < 0.01, or ^##^*P* < 0.01, 2-way ANOVA with Bonferroni post hoc test.

To account for the impact of miR-29 loss on PSC activation, we measured the α-smooth muscle (*αSMA*) activity, a well-known marker for activated PSCs, in WT and miR-29a/b1–KO mice by IHC and Western blot analyses. In WT mice, a transient increase in αSMA was observed at 2 days after caerulein dosing, and this increase gradually subsided to basal levels in 7 days (Figure 9, A and B). However, in KO mice, a dramatic increase in αSMA was noted as early as 6 hours after caerulein injection. The expression remained consistently high at 2 days, suggesting a robust increase in activated PSCs in the tissue at injury. Although reduced, prominently higher αSMA^+^-activated PSCs were detected in the KO mice during late recovery. Together, the findings suggest that loss of miR-29a/b1 promotes early and continued activation of PSCs, to accelerate fibrogenesis, thus delaying AP recovery.

To identify if PSC activation in miR-29a/b1–KO mice was mediated by *TGFβ1*, we measured active TGFβ1 in the mouse pancreata by ELISA. In saline-treated WT and KO mice, TGFβ1 levels were comparable. At 6 hours after caerulein, there was a 2- and 4-fold increase in TGFβ1 levels in WT and KO mice, respectively (Figure 9C). However, TGFβ1 went back to basal level in WT mice and continued to remain higher in the KO group for up to 7 days after final caerulein injection (Figure 9C). These observations are consistent with the sustained αSMA expression throughout AP progression, suggesting a TGFβ1-mediated activation of PSCs in miR-29a–deficient mice.

### Loss of miR-29a/b1 affects stress response in AP.

JNK is a central mediator of varied stress response pathways (31–33). Previous studies have indicated the regulatory role of JNK in caerulein-induced pancreatitis (34, 35). Therefore, we examined activation of JNK in the 2 mouse groups by measuring its phosphorylation status, where we found an increase in phospho-JNK in both the groups at 6 hours and in WT group at 2 days. Although the miR-29a/b1–KO mice exhibited increased activity of phospho-JNK at 6 hours, the activity was lower than that in the WT group (Figure 9D). In addition, examination of the activation status of c-Jun, a downstream effector of the JNK pathway, also revealed similar patterns, where an increased activity of c-Jun was observed in both the groups at 6 hours, although the activity in the KO group was significantly lower compared with the WT mice at this time point (Figure 9D). Thus, a reduced activation of the genes that accumulate as an immediate response during AP, as was observed in the KO mice, is suggestive of an insufficiency in the capacity of these animals to activate *JNK*-mediated stress response pathways during the early onset of AP.

### Systemic effects of miR-29a/b1 loss in AP.

**Figure 10:**
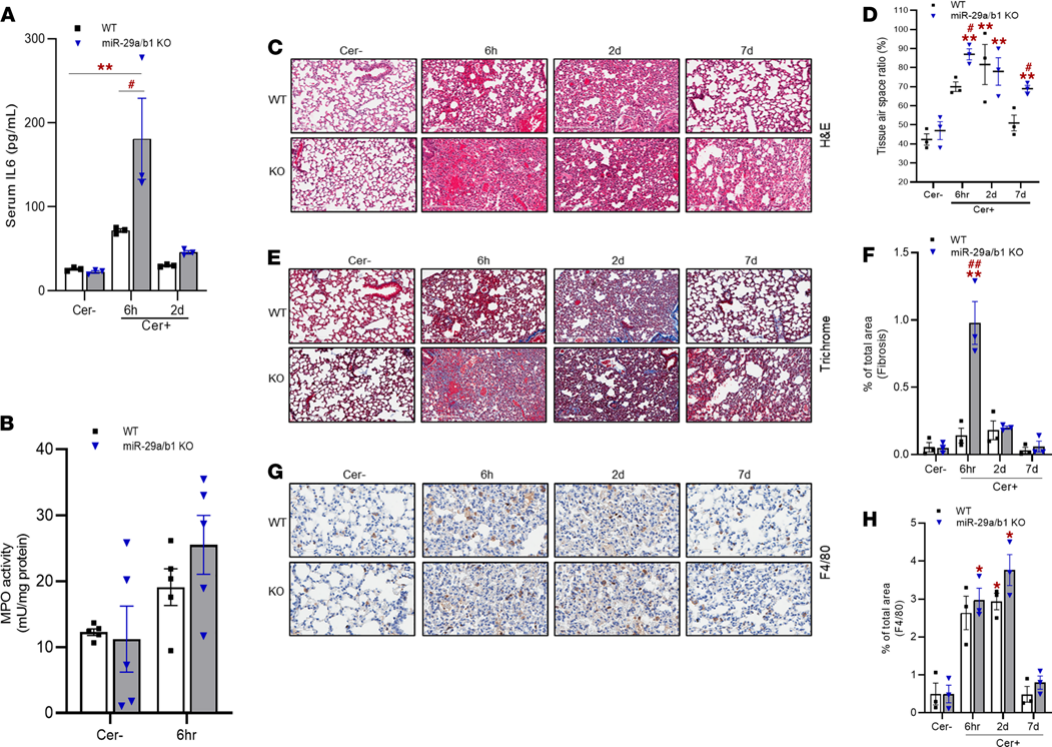
miR-29a/b1–KO exhibits systemic effects in caerulein-induced AP. (**A**) Circulating serum IL-6 levels were measured in saline- (Cer–) or caerulein-treated WT and miR-29a/b1–KO mice by ELISA (*n* = 3 mice/group/time point). (**B**) MPO activity in lung homogenates of saline- (Cer–) or caerulein-treated WT and miR-29a/b1–KO mice at the indicated time points (*n* = 5/group/time point). (**C**) Representative images for H&E staining in the lungs of saline- (Cer–) or caerulein-dosed WT and KO mice at the indicated time points. Scale bars: 200 μm. (**D**) Tissue/air-space ratio in the lung of saline- (Cer–) or caerulein-dosed WT and KO mice at the given time points (*n* = 3 animals/group/time point). (**E**) Representative images for Masson’s trichrome staining in the lungs of saline- (Cer–) or caerulein-dosed WT and KO mice at the indicated time points. Scale bars: 200 μm. (**F**) Fibrosis from Masson’s trichome staining was scored based on 5 high-powered fields. (**G**) Representative images for F4/80 staining in the lungs of saline- (Cer–) or caerulein-dosed WT and KO mice at the indicated time points. Scale bars: 50 μm. (**H**) F4/80^+^ macrophages were quantified in the lungs of WT and KO mice and represented as percentage of positive cells. IHC images and quantifications represent *n* = 3 animals/group/time point. Results in graph represent mean ± SEM. Asterisk denotes difference with saline-treated WT mice; # symbol denotes difference between caerulein dosed WT and miR-29a–KO mice at a given time point; *P < 0.05, ^#^*P* < 0.05, **P < 0.01, and ^##^*P* < 0.01, 2-way ANOVA with Šídák post hoc test.

We next assessed the systemic effects of miR-29a/b1 loss in AP. IL-6, a proinflammatory cytokine, is a known prognostic marker for AP severity (36). The serum IL-6 levels were elevated in both the groups at 6 hours, but the increase was significant in the KO mice (Figure 10A), suggesting a greater severity of the disease due to miR-29a deficiency. Although slightly elevated at 2 days, no detectable difference in IL-6 levels was found at any other time points.

Severe AP and associated inflammatory responses result in systemic complications, often with pulmonary dysfunction and acute respiratory failure (37). Thus, to assess the association of miR-29a/b1 with the systemic inflammation, we measured the pulmonary neutrophil infiltration via MPO assay. In KO mice, although slightly enhanced MPO levels were observed during the acute phase (6 hours), the values did not reach a significant threshold (Figure 10B). This was further validated by IHC analysis for MPO, where we did not observe significant neutrophil infiltration to the lung during AP injury or other time points (Supplemental Figure 3A). Histological analysis indicated a clear lung in both saline-treated WT and KO mice. At the acute phase, WT mice had congested lungs, while emphysema with a collapsed, hemorrhaged alveoli was observed in the KO mice. The lungs in both WT and KO mice appeared collapsed during the course of recovery; this condition mostly cleared by 7 days in WT, but lungs remained collapsed and congested in the KO group (Figure 10C). Concomitant to these observations, during injury, both groups had fewer alveoli, as evidenced by an increased tissue/air space ratio compared with saline-treated control mice; however, the KO mice exhibited a significantly higher ratio during the acute phase (6 hours) and at recovery (7 days) (Figure 10D). In addition, Masson’s trichrome staining showed enhanced lung fibrosis in KO mice at 6 hours after caerulein injections (Figure 10, E and F). Unexpectedly, though both the groups presented higher number of F4/80^+^ macrophages at injury, there was no difference in macrophage accumulation between the 2 groups at any time point (Figure 10, G and H). To verify that the systemic observations were due to pancreas-specific depletion of miR-29a/b1 and not a result of off-target effects in our experimental model, we measured the miR-29a levels in the lung of the KO mouse line, which showed no variation in expression from the lungs of the WT mice (Supplemental Figure 3B). Together, these data suggest that miR-29a/b1 deficiency may have moderate effects in progression of systemic complications associated with AP.

## Discussion

The current study investigated the impact of the miR-29a/b1 cluster on pancreas injury and regeneration during AP, utilizing a potentially novel conditional miR-29a/b1–KO mouse model. miR-29 plays a crucial role in fibrosis and ECM remodeling under diverse pathophysiological conditions, including that in the pancreas (8, 21, 38, 39). However, it is not known whether miR-29 regulates the fibroinflammatory mechanisms of AP. In the current study, we set out to elucidate the role of the miR-29a/b1 cluster in AP and to extensively characterize the events in miR-29a/b1–sufficient and –deficient mice through the entire course of the disease. We confirmed that mature miR-29a, generated solely from the miR-29a/b1 cluster, was lost in acinar cells, which are the major target cell type involved in AP pathogenesis. We postulated that miR-29a/b1 protects against pancreatic injury, and loss of its expression aggravates the disease pathogenesis.

Supporting our hypothesis, a robust downregulation of miR-29a was observed in the pancreata of AP mice of different etiological origins and patients with chronic pancreatitis. Serum amylase is a well-known marker for acinar cell damage, and activation of trypsin within pancreatic acinar cells is a key initiator of AP (40). Significantly enhanced amylase and trypsin activity in miR-29a/b1–KO mice at AP onset is suggestive of increased acinar damage due to miR-29a/b1 deficiency. These observations were in agreement with the H&E analyses. Markedly increased parenchymal disarray coupled with enhanced edema and acinar damage was abundant in miR-29a/b1–KO mice through the entire course of AP, unlike the WT group. In addition, delayed cell proliferation marked pancreatic injury in the KO group. Thus, it is shown from these findings that miR-29a/b1 exhibits a protective role in AP, and its loss in acinar cells intensifies AP severity and delays recovery of the injured pancreas.

We next aimed to investigate the mechanisms by which miR-29a/b1 deficiency might exacerbate AP progression. During early AP, a dysregulation in JNK activation was observed, which accounts for a deficit in stress response in the miR-29a/b1–KO mice. JNK signaling plays an essential role in apoptosis (41, 42), and acinar cell apoptosis inversely correlates with the severity of pancreatitis (43). Pancreatic acinar injury leads to inflammatory cell infiltration, neutrophil recruitment, and release of cytokines. Consistently, during the acute phase, a dramatically higher abundance of neutrophils and inflammatory cells was evident in the KO group. The mechanism by which miR-29 modulates neutrophil infiltration is not known. However, our data indicate that miR-29a/b1 may antagonize neutrophil activation and the resultant inflammatory injury at the onset of AP. Classically, neutrophils promote the pathogenesis of AP, causing acinar cell injury and local pancreatic damage (44). miR-29a/b1–KO mice showed a robust increase in *MCP1* and circulating IL-6 during the acute AP phase, promoting the local as well as systemic inflammatory response. Additionally, aberrant patterns of macrophage populations in the pancreas were also detected in the KO mice. Macrophages are important determinants of AP severity. Though at 6 hours after caerulein injection, comparable macrophage infiltrates were detected in both groups, in WT mice, these cells quickly disappeared during early recovery. But these cells were abundant and persistent in the KO group until late recovery. It is possible that miR-29a/b1 depletion has an impact on MCP1-mediated transmigration of monocytes/macrophages during AP. Furthermore, depletion of miR-29a/b1 escalated CD68^+^ M1 differentiation at early AP onset, aggravating local inflammation, which relates with the extensive acinar damage observed in the KO mice. Downregulation of the CD163^+^ M2 population during the acute phase further supports this notion. Consistent repression of CD163^+^ M2 cells may promote the continued fibrosis and tissue damage observed in these mice. Though no definite trend was observed in WT mice, the transient downregulation of the M2 population in this group may be attributed to the rapidly changing states of activation of the macrophages or their heterogenous population in response to local inflammation. Nonetheless, it is clear from these results that miR-29 plays a crucial role in the early inflammatory mechanisms of AP and engages in modulating the severity of the disease. miR-29a/b1 deficiency resulted in profound ECM accumulation throughout AP progression, with maximum ECM accumulation at 2 days after injections. Excess and continued deposition of ECM proteins such as collagen and fibronectin resulted in a significant delay in AP recovery in the KO mice. miR-29a directly targets several essential fibrillar and nonfibrillar ECM proteins associated with tissue maintenance and pancreas remodeling, thereby alleviating fibrogenesis (11, 21, 39). The dramatic and sustained increase in matrix deposition in the KO AP mice may be indicative of a direct miR-29 regulation of its ECM targets. In addition, the enhanced injury at the early AP onset, characterized by deficiency in stress response pathways such as JNK signaling in the miR-29a/b1–KO mice, might also contribute toward the prolonged and aberrant fibroinflammatory responses, via one or more indirect mechanisms, together deterring parenchymal tissue repair and increased ECM accumulation at the later phases of the disease progression.

Activated PSCs are the key modulators of ECM turnover and pancreatic fibroinflammatory mechanisms during pancreatitis (30, 45). PSCs were transiently activated in the WT AP mice at an early phase (2 days) but disappeared during recovery. While in miR-29a/b1–KO mice, activated PSCs were abundant through the entire AP time course. This suggests an essential role of miR-29a/b1 to halt PSC activation and maintenance of their quiescent states. Interestingly, we observed a similar role of miR-29a in regulation of PSCs in the context of PDAC matrix remodeling (8). Activation of PSCs is marked by upregulation of a plethora of cytokines, including IL-6, IL-10, and TGFβ1 and ECM proteins such as collagens, laminins and metallopeptidases (30, 46). Thus, abundant and continuous activation of PSCs in KO mice might be one of the plausible explanations for the aberrant matrix deposition during AP. Of note, because miR-29a expression was lost in acinar cells but not PSCs in our miR-29a/b1–KO mouse model, the activation of PSCs or associated ECM deposition might be the result of altered signaling from damaged acinar cells in these mice. Moreover, in the KO mice, TGFβ1 was persistently upregulated through the entire AP course, unlike in WT mice, where the upregulation was transient. This further suggests that the persistence of activated PSC in the KO mice might be the consequence of altered TGFβ1 signaling. Together, these findings depict a regulatory role of miR-29a/b1 in PSC-mediated mechanisms of AP.

Collectively, the data presented in the current study depict for the first time to our knowledge that miR-29a/b1 is critical in AP pathogenesis. AP is characterized by general loss of miR-29a, which triggers the activation of a cascade of interconnected mechanisms during the disease progression. It is also evident from our findings that PSCs play key roles in miR-29–mediated AP regulation. These findings strongly suggest that restoration of miR-29a early on in AP may ameliorate the fibroinflammatory aberrations and reinstate the balance in ECM turnover, critical for pancreatic tissue remodeling and restoration of organ architecture. One of the limitations of this study is that our observations are based on the effects of loss of miR-29a/b1 in vivo. Future studies should be designed to assess the impact of gain-of-function of miR-29a/b1 in AP in vivo, which can be achieved by orthotopic delivery of the molecule in mouse pancreata. In addition, with the current findings, it would be essential to investigate the precise signaling cascades and the associated network under the regulation of miR-29a/b1 that influence acinar-PSC crosstalk in AP, where single-cell RNA sequencing analysis approach might be advantageous.

Taken together, our findings provide insights into the regulation of miR-29a/b1 in AP pathobiology, opening the possibilities for novel clinical approaches to target a multitude of fibroinflammatory circuits by utilizing the molecule. Future comprehensive investigations to delineate the molecular mechanisms regulated by miR-29a/b1 in AP will aid in developing therapeutic strategies to prevent or reverse the disease advancement.

## Methods

### Development of miR-29a/b1–floxed mice.

Floxed mice for the miR-29a/b1 genomic cluster were generated using the CRISPR/Cas9 system (Figure 2). In brief, 2 floxed sites (LoxP) flanking the miR-29a/b1 cluster were introduced via homology-directed repair (HDR) using a single-stranded oligodeoxynucleotide (ssODN) donor repair template consisting of 5′ and 3′ LoxP sites, Ecor1 restriction site, and short homology arms to flank endogenous alleles (Supplemental Table 3 and Figure 2A). To induce double-stranded breaks (DSB) in the miR-29a/b1 flanking regions and facilitate HDR with the ssODN, 2 guide RNAs (gRNAs) that selectively bind to the 5′ and 3′ miR-29a/b1 genomic regions (Table 1) were identified for target cutting efficiency, as well as potential off target candidates using the CHOPCHOP (47). An in vitro cleavage assay was used to validate both the identity and estimated cleavage efficiency of a PCR amplified fragment containing the guide target region (Figure 2B). On the day of microinjection, CRISPR RNA (crRNA) was paired with transactivating CRISPR RNA (tracrRNA) as a 2-part gRNA and complexed with Cas9 protein to form a ribonucleic protein (RNP) (Integrated DNA Technologies). Subsequently, pronuclear microinjection of 10 ng/μL RNP and 10 ng/μL ssODN mixed in microinjection buffer (10 mM TRIS, 0.1 mM EDTA) were administered to the C57BL/6N mouse (The Jackson Laboratory) zygote pronuclei via constant positive flow injection, followed by embryo transfers into pseudo pregnant C57BL/6N recipients. Founder animal genomic DNA was isolated using 2D bar-coded tracking on an integrated and LIMS tracked Hamilton robot using DNAdvance magnetic bead chemistry (Beckman Courtier) and screened for the presence of each LoxP site via TaqMan based qPCR (Figure 2C and Table 2). Subsequently, PCR was performed with the double LoxP^+^ samples using primers designed to bind outside of the homology arm and the product was sequenced (Table 1 and Supplemental Tables 4 and 5) to confirm sequence fidelity and on target confirmation of the LoxP sites. Founder (P0) and N1 mice were backcrossed to C57BL/6N for the generation of N1 and N2 mice heterozygous for the correct HDR allele. N2 mice were bred with C57BL/6 to maintain mouse line or crossed with Pdx1-Cre to generate miR-29a/b1–KO mice as described below. Primers and gRNA sequences for the CRISPR assays are detailed in Table 1.

### Generation of miR-29a–KO mice.

As depicted in Figure 3A, miR-29a/b1–KO transgenic mice were generated by crossing miR-29a/b1–floxed mice with a Pdx1-Cre (*Cre^+/+^*) driver line that expresses Cre recombinase under the pancreas-specific promoter Pdx1, allowing deletion of the miR-29a/b1 allele in pancreas. Crossing homozygous mice for miR-29a/b1–floxed allele (*miR-29ab1^–/–^*) with Pdx1-Cre^+/+^ mouse line yielded *miR-29ab1^+/–^*; *Pdx1-Cre^+/–^* haplotypes in the F1 generation. Backcrossing these F1 mice with miR-29a/b1–floxed mice generated mice in the F2 generation, with 25% mice having the experimental genotype (*miR-29ab1^–/–^*; *Pdx1-Cre^+/–^*). Age-matched *miR-29ab1^+/+^*; *Pdx1-Cre^–/–^* littermates or C57BL/6 mice were used as WT. All mice were housed in a pathogen-free standard facility at IU with a 12-hour light/dark cycle, controlled temperature and controlled humidity, and they had access to food and water ad libitum.

### Genotyping.

DNA for genotyping was obtained from mouse tails (0.2 cm) snipped at 3 weeks of age. Tail snips were incubated in 150 μL of tail lysis buffer consisting of 50 mM NaOH and 2 μM EDTA at 95°C for 1 hour, followed by neutralization with 10 μL of 1M Tris. In total, 100 μL of supernatant was collected after spinning down to obtain DNA for PCR reactions.

Cre and LoxP genotypes of the offspring were determined by a PCR strategy depicted in Figure 3, A and B. Two oligonucleotides that bind upstream of a LoxP site (LoxP forward) and downstream of the LoxP site (LoxP reverse) were designed to discriminate between the WT and floxed alleles on genomic DNA (Table 3). For WT (*miR-29ab1^+/+^*) and homozygous floxed (*miR-29ab1^–/–^*) mice, the expected fragment sizes are 141 and 181 bp, respectively, while for heterozygous (*miR-29ab1^+/–^*) mice, both 141 and 181 bp fragments were detected simultaneously. Pancreas-specific miR-29a–KO (*miR-29ab1^–/–^*; *Pdx1-Cre^+/–^*) mice were identified based on Cre positivity, detected by PCR analysis to produce a 400 bp amplicon using Cre forward and Cre reverse primers, along with the presence of WT and/or floxed LoxP alleles (Table 3 and Figure 3B). The genotype was further validated by qPCR analysis for miR-29 in the mice pancreata at their necropsies (Figure 3C).

### Experimental models of AP.

AP was induced to WT and miR-29a/b1–KO adult mice (6–8 weeks of age) by (a) 8 hourly i.p. injections of sterile caerulein (Sigma-Aldrich) for 2 consecutive days (50 μg/kg body weight solubilized in PBS) or by (b) 2 i.p. injections of sterile L-arginine monohydrochloride (Sigma Aldrich) prepared in PBS (8%), pH adjusted to 7.0, at a dose of 4 g/kg body weight.

Control (WT and KO) mice received an equal amount of sterile saline (PBS). Caerulein/PBS-injected mice were sacrificed at different time points following the final dose: 6-hour (acute phase), 2-day or 4-day (early recovery phase), and 7-day or 10-day (late recovery phase) for serum and tissue collection. Mice injected with L-arginine/PBS were sacrificed at 72 hours after the first injection. Each group of mice consisted of 5–7 randomly selected littermates per time point/genotype/group.

Pancreatic specimens for mice fed with ethanol-rich diet and corresponding normal diet were provided by Aurelia Lugea, Cedar-Sinai Medical Center.

### miR-29a expression in patients with chronic pancreatitis.

miR-29a expression in the pancreases of patients with chronic pancreatitis and patients with normal donor pancreases were accessed from a publicly available GEO data set (GSE24279).

Pancreatic tissue specimens from patients with chronic pancreatitis and those with normal adjacent pancreatic tissue sections were obtained from the Indiana University Cancer Center Tissue Bank.

### IHC.

Pancreata and lungs from the euthanized mice at each time point were fixed immediately in 10% formalin; they were then paraffin embedded and sectioned, and IHC staining was performed as described previously (21). The primary antibodies used are enlisted in Table 4. Images were acquired using an Aperio whole slide imaging system (Aperio ScanScope System). The slides were reviewed and evaluated by an experienced pathologist in a blinded fashion. For each animal, inflammatory cell infiltration was evaluated in 5 random visual fields at ×200 magnification. A scale (0, negative; 0.5, minimal; +1, mild; +2, moderate; +3, strong; +4, severe) was used to quantify inflammation based on the density of inflammatory cell (neutrophils, monocytes/macrophages, and lymphocytes) infiltrates. Quantification for all other protein markers and fibrosis (Masson’s trichrome staining) was performed on 5–7 random, nonoverlapping sections at ×200 magnification using ImageJ software (NIH) and was expressed as percentage (%) stained area per total area examined.

Fluorescence staining of the tissue sections was performed as previously described (48). The tissues were probed with the following primary antibodies: α-amylase (3796S, Cell Signaling Technology), insulin (4590S, Cell Signaling Technology), and CK19 (ab52625, Abcam), followed by secondary staining with Alexa Fluor 488 goat anti–rabbit IgG (A11008, Invitrogen). Slides were mounted with Vectashield mounting medium with DAPI (H-1200, Vector Laboratories) and exposed to DAPI and FITC filters, and the images were superimposed.

### RNA extraction and qPCR.

Total RNA was extracted from snap-frozen tissue samples using Trizol Reagent (Invitrogen) following manufacturer’s instructions. The concentration and purity of the extracted RNAs were measured using a Nanodrop 2000 Spectrophotometer (Thermo Fisher Scientific).

RNA was reverse transcribed to cDNA using High-capacity cDNA Reverse Transcription kit (4368814, Thermo Fisher Scientific) with random primers for genes or custom primer pool for miRNA. To measure mature miR-29a, -29b, and -29c expression, reactions were set up utilizing TaqMan Fast Advanced Master mix (catalog 4444557), with TaqMan probe and primers for mature miR-29a (478587_mir), miR-29b (478369_mir), miR-29c (479229_mir), or U6 snRNA (001973) from Thermo Fisher Scientific. For quantification of gene expressions, qPCR was performed with PowerUp SYBR Green Mastermix (A25742, Thermo Fisher Scientific), and custom primers (Table 3) were generated by Integrated DNA Technologies. miRNA and mRNA expressions were normalized to U6 and ACTB, respectively. Samples were run in duplicate in a total volume of 10 μL on an ABI 7500 Real-time PCR machine (Applied Biosystems). Relative expression was analyzed using the ΔΔCT method.

### Western blot.

Pancreas tissues were homogenized on ice in lysis buffer (125 mM tris-HCl [pH 6.8], 4% SDS, 4M urea) with 1× protease inhibitor cocktail (87786, Thermo Fisher Scientific), and supernatant was collected following centrifugation at 10,000*g* for 20 minutes at 4°C. Protein concentrations were determined using BCA Protein Assay Kit (23225, Pierce Biotechnology). Western blots were then performed as described previously (21). Densitometry analysis was performed using ImageJ software to quantify protein bands, which were then normalized against loading control GAPDH. The primary antibodies used in this study were anti-COL1A1 (NBP1-30054, Novus Biologicals), anti-fibronectin (NBP1-91258, Novus Biologicals), phospho–c-Jun (ab32385, Abcam), phospho-JNK (AF1205, Novus Biologicals), and anti-GAPDH (MA5-15738, Thermo Fisher Scientific).

### PSC isolation.

PSCs were isolated from WT and miR-29a/b1–KO mice based on a previously described protocol (49) with some modifications. Pancreatic tissue from each mouse was isolated and partially digested in 0.13% collagenase P (MilliporeSigma), 0.09% protease (Thermo Fisher Scientific), and 0.175% DNAse (NEB) in NaCl^+^ Gey’s balanced salt solution (GBSS; MilliporeSigma) by injecting the enzyme mix with caerulein syringes followed by incubation at 37°C for 7 minutes. The partially digested pancreas was thereafter transferred to a petri dish and minced finely with removal of any contaminant tissues, which were then incubated at 37°C for another 7 minutes. The digested tissue was then filtrated using a 250 μm nylon mesh, and the filtrate was centrifuged at 450*g* at 4°C for 10 minutes. Supernatant was removed, and the pellet was washed in GBSS + NaCl with 3% BSA and recentrifuged at 450*g* at 4°C for 10 minutes. The resultant pellet was resuspended in GBSS + NaCl with 3% BSA (Thermo Fisher Scientific), to which 28.7% Nycodenz solution (Crescent Chemical Co Inc.) in GBSS + NaCl was added and mixed well. In a round-bottom polycarbonate centrifuge tube, 6 mL of GBSS + NaCl consisting of 0.3% BSA solution was added. The cell suspension after centrifugation was carefully layered underneath the aqueous BSA solution using a 30 mL syringe attached to a polyvinyl pipette without disrupting the interface. The solution was centrifuged at 1400*g* for 20 minutes at 4°C. The fuzzy band above the interface containing the PSCs was then carefully separated and washed with GBSS + NaCl with 0.3% BSA, and it was centrifuged at 450*g* for 15 minutes at 4°C. The pellet was resuspended in Iscove’s modified Dulbecco’s medium (IMDM; Thermo Fisher Scientific) consisting of 10% FBS, 4 mM glutamine (Thermo Fisher Scientific), 100 U/mL penicillin (Thermo Fisher Scientific), and 100 μg/mL streptomycin (Thermo Fisher Scientific). After counting, cells were plated in 6-well plates or 25 cm^2^ flasks and cultured in a humidified incubator at 37°C with 5% CO_2_ to confluence.

### Acinar cell isolation.

Acinar cells were isolated following a protocol described previously (50). Briefly, pancreata from WT and miR-29a/b1–KO groups were isolated, washed immediately, transferred to HBBS (Thermo Fisher Scientific), sliced into small pieces, and centrifuged at 450*g* for 2 minutes at 4°C. Supernatant was removed, and pancreas sections were suspended in collagenase IA solution (1× HBSS, 10 mM HEPES, 200 U/mL collagenase IA, and 0.25 mg/mL trypsin inhibitor; MilliporeSigma); they were incubated for 20–30 minutes at 37°C, with dissociation of the sections with serological pipettes every 5 minutes. The dissociated tissue was then washed with washing buffer (1× HBSS, 5% FBS, and 10 mM HEPES) and centrifuged at 450*g* for 2 minutes at 4°C. Supernatant was aspirated, and the pellet was washed with washing buffer and recentrifuged at 450*g* for 2 minutes at 4°C three times. The pellet was then resuspended in Waymouth’s Medium (Thermo Fisher Scientific) supplemented with 2.5% FBS, 1% penicillin-streptomycin, 0.25 mg/mL trypsin inhibitor (MilliporeSigma), and 25 ng/mL recombinant human Epidermal Growth Factor (MilliporeSigma). Cells were then filtered through a 100 μm filter. The filtrate containing the acinar cells was rinsed in the supplemented Waymouth’s Medium, counted, seeded in a 6-well petri plate, and cultured in suspension in a humidified incubator at 37°C with 5% CO_2_. After 24 hours, the suspended acinar cells were transferred into a new 6-well plate coated with rat tail type I collagen (Thermo Fisher Scientific) and cultured at the same conditions to confluence.

### Islet isolation.

Islets were isolated following a protocol described previously (51). Briefly, WT and miR-29a/b1 mice were euthanized by cervical dislocation; pancreata were inflated with slow delivery of a mixture of protease and collagen, administered utilizing a cannula. The inflated pancreas was then removed and placed in a 50 mL tube with HBSS P/S solution (1× HBSS + 1% penicillin/streptomycin) and allowed to dissociate by incubation into a 37°C water bath for 15 minutes with occasional swirling. Thereafter, 25–30 mL HBSS/BSA solution (1× HBSS + 1% penicillin/streptomycin + 0.3% BSA; Thermo Fisher Scientific) was added to the tube, which was then centrifuged at 290*g* for 1 minute. The resulting supernatant was aspirated, and 10 mL of HBSS/BSA solution was added to the tube with aspiration using a 30 mL syringed attached to a 14 G needle. The solution was filtered with a plastic tea strainer and collected in a 50 mL tube. This was followed by rinsing with HBSS/BSA solution, centrifugation at 330*g* for 2 minutes at room temperature, and aspiration of the resulting supernatant. In total, 10 mL of cold 1100 histoplaque (MilliporeSigma) was added to each tube, which was overlayed with 10 mL HBSS/BSA solution and centrifuged at 900*g* for 18 minutes. The resultant supernatant consisting of the isolated islets was removed using a large-bore 25 mL pipet and passed through an inverted 70 μm filter. The islets were rinsed into a 10 cm petri plate by pipetting 10 mL media (RPMI + 10% FBS + 1% glutamine + 1% penicillin/streptomycin) through the filter. The islets were cultured in suspension in a humidified incubator at 37°C with 5% CO_2_.

### Measurement of serum amylase, trypsin, and MPO enzymatic activity.

Whole blood samples collected in anticoagulant treated tubes (450472, Greiner Bio-one) at necropsy were centrifuged at 2000*g* for 10 minutes at 4°C, and the resulting supernatant (serum) was collected for downstream analyses. Amylase activity in these mouse serum samples was measured commercially by IDEXX Bioanalytics using optimized protocol.

Trypsin enzymatic activity was measured from pancreas homogenates using trypsin activity assay kit (3043, Chondrex Inc.) following manufacturer’s instructions. Briefly, small pancreatic tissue pieces (25–30 mg) were washed in PBS, homogenized in 1× RIPA lysis buffer (ab156034, Abcam), and centrifuged at 13,000*g* for 10 minutes at 4°C. The total protein concentration from the collected supernatant was measured by BCA analysis (23225, Pierce Biotechnology), which was then used for the trypsin activity assay.

Neutrophil infiltration in the pancreas and lungs was quantified by measuring the MPO activity in the tissues utilizing the MPO Activity Assay Kit (K747, BioVision). For this assay, tissue samples (~20 mg) washed in PBS were homogenized in 500 μL of MPO Assay Buffer at 10,000*g* for 10 minutes at 4°C. Concentration of total protein from the collected supernatant was estimated by BCA analysis (23225, Pierce Biotechnology) and then utilized to measure MPO activity following manufacturer protocol.

### Total collagen quantification.

Total collagen in the pancreatic tissues was estimated by measuring the hydroxyproline content utilizing the hydroxyproline assay kit (6017, Chondrex Inc.) following manufacturer protocol. Briefly, 10 mg of pancreatic tissue was homogenized, and the sample homogenate was hydrolyzed in 10N HCl at 120°C for 24 hours. The samples were then neutralized, and hydroxyproline was oxidized by incubating with 1× chloramine T solution for 20 minutes, followed by an incubation with 1× dimethyl amino benzaldehyde (DMAB) solution diluted with perchloric acid at 60°C for 30 minutes. Absorbances were measured at 530 nm, and the hydroxyproline content was measured by comparison with the values from samples with known concentrations utilizing a standard curve.

### Quantification of IL6 and TGFβ1.

Serum IL-6 and TGFβ1 levels in the pancreas were determined using commercially available ELISA kits following manufacturer’s instructions for IL-6 (KE10007, Proteintech) and TGFβ1 (DY1679, R&D Systems). Serum for IL-6 quantification was obtained from collected blood of the mice at necropsy. For measuring TGFβ1 levels, pancreatic tissues were homogenized in PBS with 1× protease inhibitor cocktail (87786, Thermo Fisher Scientific), and supernatant was collected by centrifugation at 10,000*g* for 10 minutes at 4°C. TGFβ1 in the protein extracts was activated by incubation with 0.25N HCl, followed by neutralization with 0.25N NaOH/0.125M HEPES, and it was then used for the ELISA assay.

### Statistics.

All data are expressed as mean ± SEM from at least 3 animals per group for each time point. AP induction and analyses were performed for 3 independent trials. GraphPad Prism (v9) and Microsoft Excel were used for statistical analyses. Statistical analysis between 2 groups was performed using 2-tailed Student’s *t* test for pairwise comparison, 1-way ANOVA with Dunnett’s or Tukey’s post hoc tests, or 2-way ANOVA with Bonferroni or Šídák post hoc tests for multiple comparisons. Differences were considered statistically significant at *P* < 0.05, indicated with asterisks or pound. *P < 0.05 or ^#^*P* < 0.05; **P < 0.01 or ^##^*P* < 0.01.

### Study approval.

All mouse protocols were reviewed and approved by the IU Animal Care and Use Committee. *Guide for the Care and Use of Laboratory Animals* (National Academies Press, 2011) was followed for all animal housing, use, and euthanasia procedures.

## Author contributions

JK and SD conceptualized and designed the studies; SD, LMU, PR, and JJK performed experiments and acquired data; BW laid the foundation and collaborated in the generation of the experimental miR-29a–floxed mice; SD and JK analyzed and interpreted the data; SP, JJE, and ELF provided with critical resources for the study; SD drafted the manuscript; SD, JJK, SP, JJE, and JK revised the manuscript critically with important intellectual content. All authors approved the final version of the manuscript.

## Supplementary Material

Supplemental data

## Figures and Tables

**Table 1:**
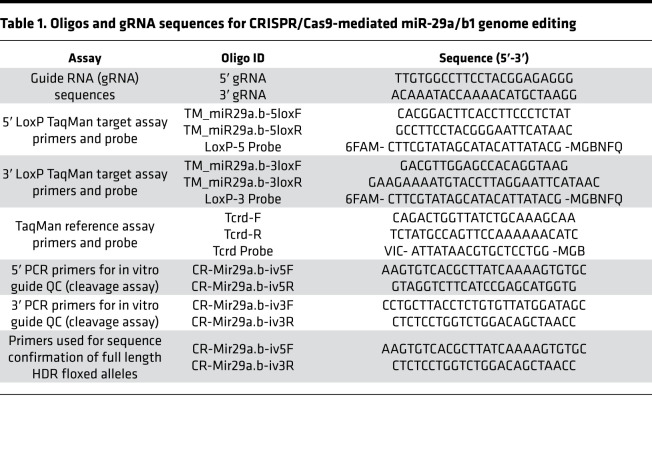
Oligos and gRNA sequences for CRISPR/Cas9-mediated miR-29a/b1 genome editing

**Table 2:**
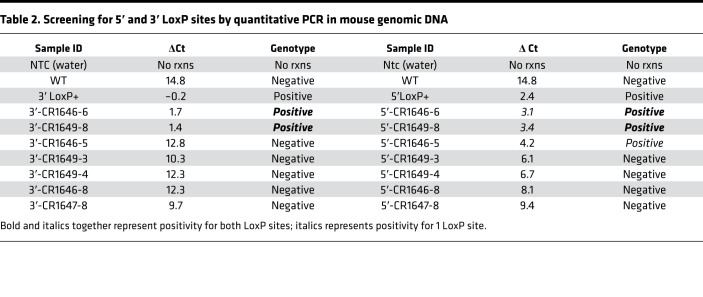
Screening for 5′ and 3′ LoxP sites by quantitative PCR in mouse genomic DNA

**Table 3:**
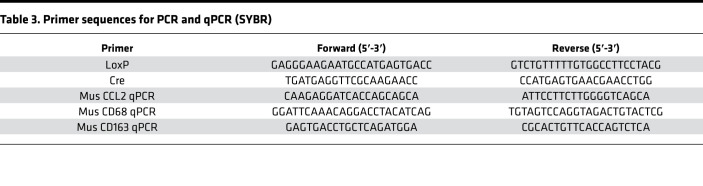
Primer sequences for PCR and qPCR (SYBR)

**Table 4:**
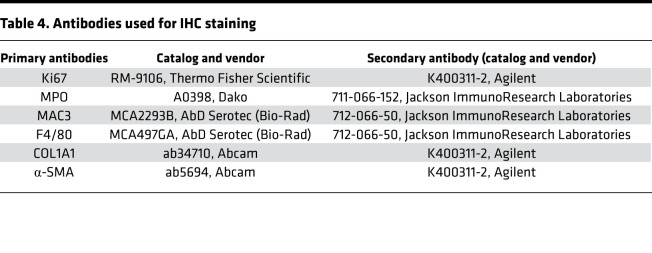
Antibodies used for IHC staining

## Version 1 08/31/2021

In-Press Preview

## Version 2 10/08/2021

Electronic publication
