# Supplementary material for: Loss of miR-29a/b1 promotes inflammation and fibrosis in acute pancreatitis
Source: JCI Insight. 2021 Oct 8;6(19):e149539. doi: 10.1172/jci.insight.149539 (PMC8525644; doi:10.1172/jci.insight.149539)
Supplement: Supplemental data [file jciinsight-6-149539-s239.pdf]

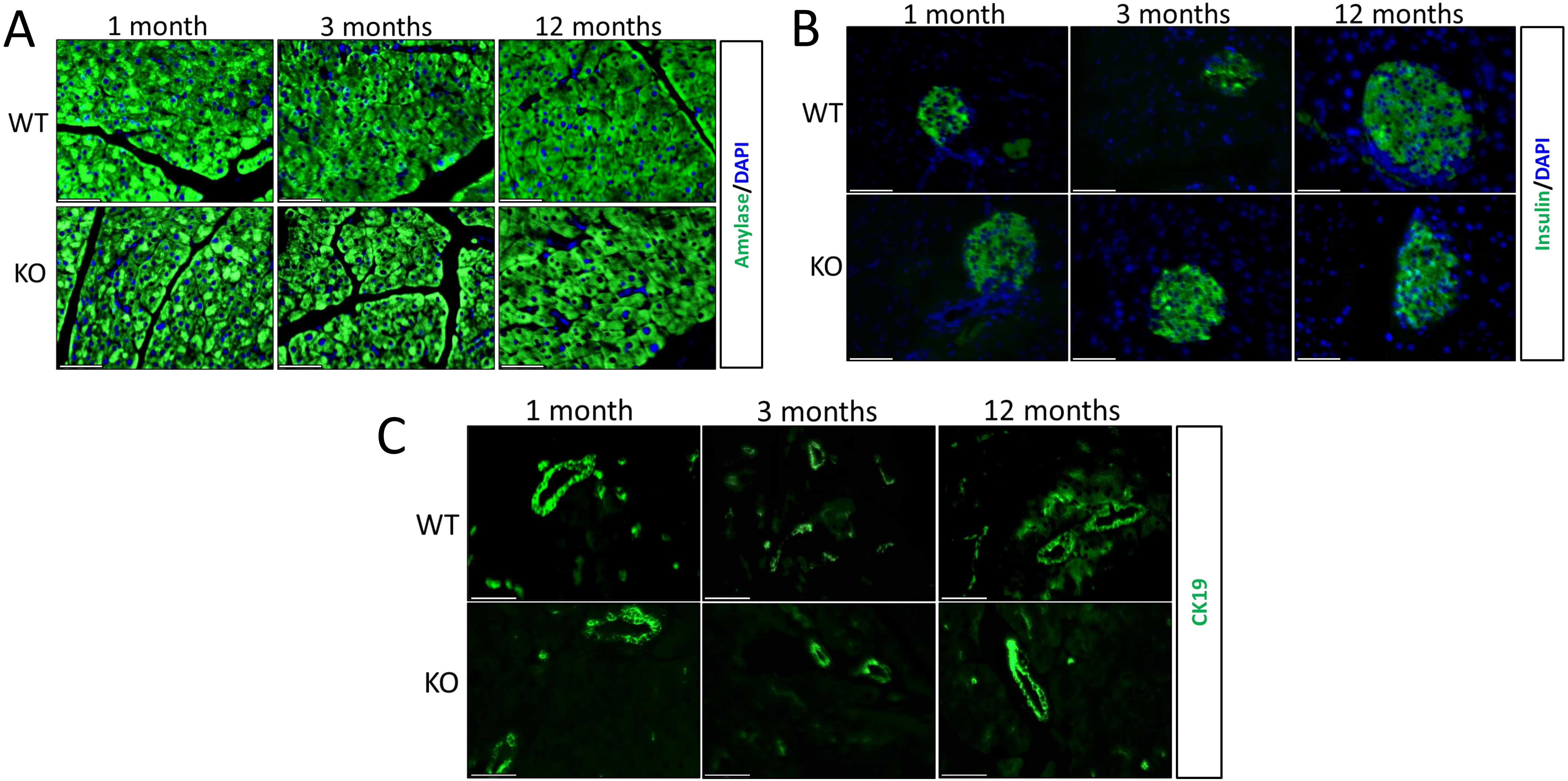

Supplemental Figure 1. Pancreatic sections of WT and KO mice at 1, 3 and 12 months of age immunostained with antibodies against **(A)** amylase (green)/ counterstained with DAPI (blue), **(B)** insulin (green)/counterstained with DAPI (blue), and **(C)** CK19 (green), photographed at 40X objective (scale bar= 62.2 µm).

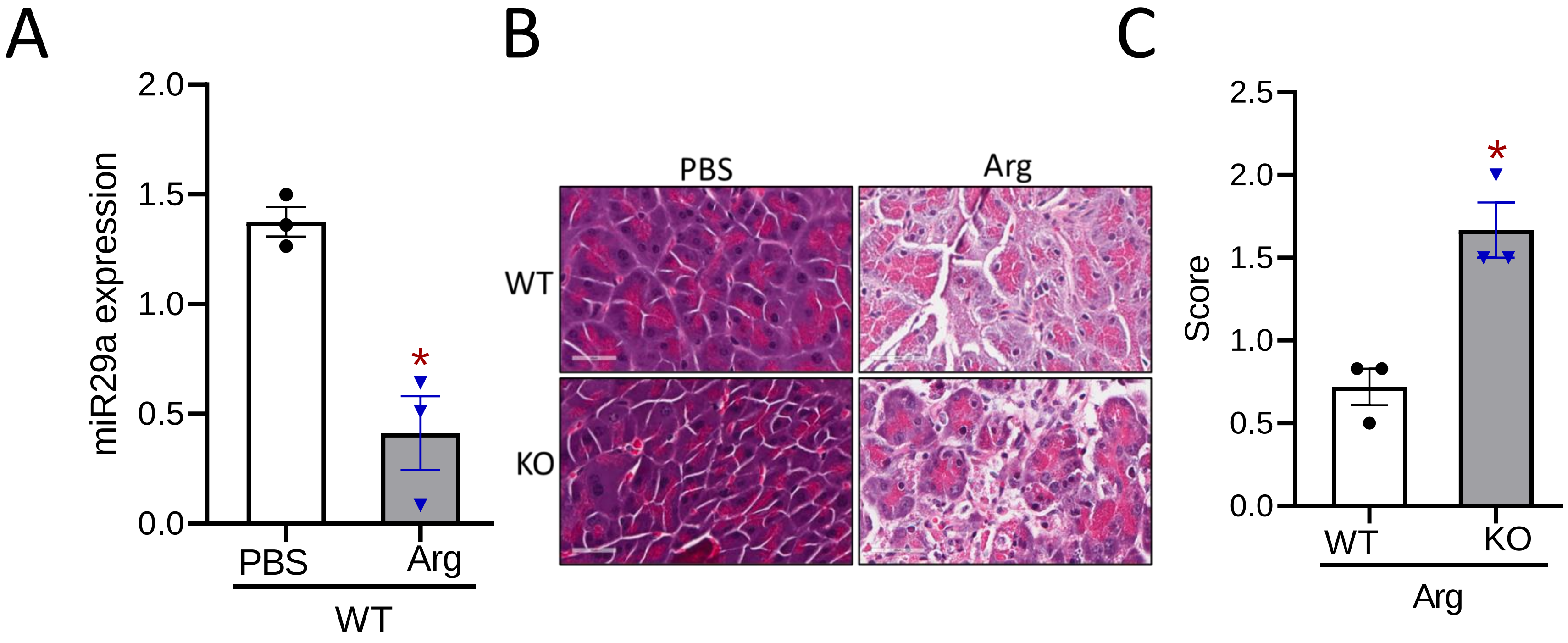

Supplemental Figure 2. (A) qPCR analysis of miR-29a expression in pancreata of WT mice 72h post final L-arginine injection. **(B)** Representative H&E images of pancreatic sections of WT and KO mice treated with PBS or L-arginine at 72h post L-arginine administration (scale bars = 40 µm). **(C)** Quantification of inflammatory cell infiltration from H&E-stained pancreatic sections of L-arginine dosed WT and miR-29a/b1 KO mice 72hrs post final injection. Graphs represent Mean ± SEM; n=3 mice/group/timepoint, *p< 0.05, 2-tailed Student's *t* test.

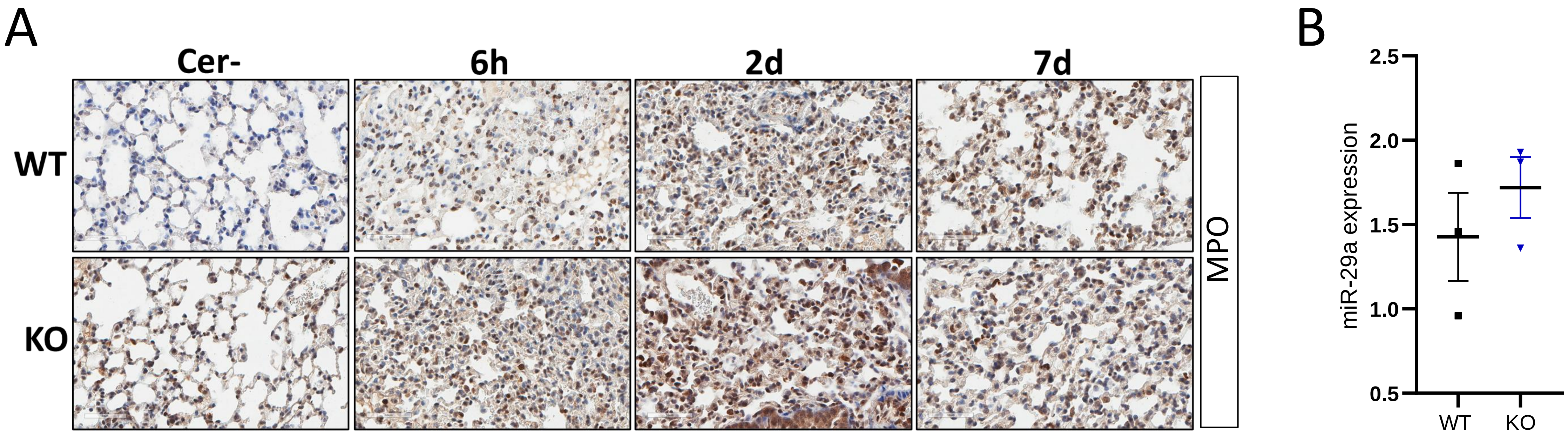

Supplemental Figure 3. (A) Representative images for MPO staining in the lungs of saline (Cer-) or caerulein dosed WT and KO mice at the indicated time-points (Scale bars = 50 µm)**. (B)** miR-29a expression in the lungs of WT and miR-29a KO mice assessed by qPCR analysis. Results represent Mean ± SEM; n= 3mice/group/timepoint, 2-tailed Student's *t* test.

Supplemental Table 1. Summary of clinical characteristics of human subjects.[1]

	Normal	Chronic Pancreatitis (CP)
Pancreas		
Normal	4	0
Pancreatitis	0	4
Sex		
Male	2	3
Female	2	1
Average age at procedure	68.75	66.75
Age Range	61-77	56-76
Race		
Non-Hispanic, White	4	4

[1] Source: Indiana University Simon Cancer Center Tissue Bank and Department of Pathology

Supplemental Table 2. Demographic and clinical characteristics of normal and human chronic pancreatitis patients.[1]

ID	Sex	Birth Year	Ethnicity	Race	Procedure Year	Histological Characteristics of pancreas
NP-1	F	1932	Non-Hispanic	White	2006	100% normal pancreas
NP-2	M	1947	Non-Hispanic	White	2010	100% normal pancreas
NP-3	F	1951	Non-Hispanic	White	2012	100% normal pancreas
NP-4	M	1936	Non-Hispanic	White	2013	100% normal pancreas
CP-1	M	1961	Non-Hispanic	White	2010	100% pancreatitis
CP-2	M	1949	Non-Hispanic	White	2010	100% pancreatitis
CP-3	M	1955	Non-Hispanic	White	2011	100% pancreatitis
CP-4	F	1935	Non-Hispanic	White	2011	100% pancreatitis

[1] Source: Indiana University Simon Cancer Center Tissue Bank and Department of Pathology

Supplemental Table 3. miR-29a/b1 KO Strategy using CRISPR/CAS9.

Long ssODN with offset homology:

Color codes: Blue: 5’-homology; Green: loxP; Purple: EcoR1; Black: Floxed miR-29a/b1 genomic region; Red: 3’-homology.

```
ccccacccccacctgctagatgaggtcagcataggatcgcctgaaattaggaactattgcacggacttcaccttccctctataacttcgtatagcatacattatacgaagttatGAATTCccg
taggaaggccacaaaaacagacgacaaagcttcttcaggaagctggtttcatatggtggtttagatttaaatagtgattgtctagcaccatttgaaatcagtgttcttggtggagaactacttcgc
tgccgaccgcacaaagaaatgagtgtacagccctgaagtaagtgtccagtgtctaaaacaggctaccagagcctgccctcgcccaccctgcttacctctgtgttatggatagctgattagtcaac
caccgttagaattgcatacttgttcatgtaataagccttctctggaagtggactccaccatgctcggatgaagacctacatatacgacagattgaaggcctgggccttcccagtgcacatgacctc
ttgtgaccccttagaggatgactgatttcttttggtgttcagagtcaatagaattttctagcaccatctgaaatcggttataatgattggggaagagcaccgttcagctgactacgttattgctgacg
ttggagccacaggtaagaattaagaaacaaataccaaaacatgataacttcgtatagcatacattatacgaagttatGAATTCctaaggtacattttcttcctatcttaaacttgactttcat
```

Genomic sequence of miR-29a/b1 (yellow highlight)

Cut

```
9601   CTTCACCTTC CCTCTCCGTA GGAAGGCCAC AAAAACAGAC GACAAAGCTT CTTCAGGAAG CTGGTTTCAT ATGGTGGTTT AGATTTAAAT AGTGATTGTC
       GAAGTGGAAG GGAGAGGCAT CCTTCCGGTG TTTTTGTCTG CTGTTTCGAA GAAGTCCTTC GACCAAAGTA TACCACCAAA TCTAAATTTA TCACTAACAG
9701   TAGCACCATT TGAAATCAGT GTTCTTGGTG GAGAACTACT TCGCTGCCGA CCGCACAAAG AAATGAGTGT ACAGCCCTGA AGTAAGTGTC CAGTGTCTAA
       ATCGTGGTAA ACTTTAGTCA CAAGAACCAC CTCTTGATGA AGCGACGGCT GGCGTGTTTC TTTACTCACA TGTCGGGACT TCATTCACAG GTCACAGATT
9801   AACAGGCTAC CAGAGCCTGC CCTCGCCCAC CCTGCTTACC TCTGTGTTAT GGATAGCTGA TTAGTCAACC ACCGTTAGAA TTGCATACTT GTTCATGTAA
       TTGTCCGATG GTCTCGGACG GGAGCGGGTG GGACGAATGG AGACACAATA CCTATCGACT AATCAGTTGG TGGCAATCTT AACGTATGAA CAAGTACATT
9901   TAAGCCTTCT CTGGAAGTGG ACTCCACCAT GCTCGGATGA AGACCTACAT ATACGACAGA TTGAAGGCCT GGGCCTTCCC AGTGCACATG ACCTCTTGTG
       ATTCGGAAGA GACCTTCACC TGAGGTGGTA CGAGCCTACT TCTGGATGTA TATGCTGTCT AACTTCCGGA CCCGGAAGGG TCACGTGTAC TGGAGAACAC
.0001  ACCCCTTAGA GGATGACTGA TTTCTTTTGG TGTTCAGAGT CAATAGAATT TTCTAGCACC ATCTGAAATC GGTTATAATG ATTGGGGAAG AGCACCGTTC
       TGGGGAATCT CCTACTGACT AAAGAAAACC ACAAGTCTCA GTTATCTTAA AAGATCGTGG TAGACTTTAG CCAATATTAC TAACCCCTTC TCGTGGCAAG
.0101  AGCTGACTAC GTTATTGCTG ACGTTGGAGC CACAGGTAAG AATTAAGAAA CAAATACCAA AACATGCTAA GGTACATTTT CTTCCTATCT TAAACTTGAC
       TCGACTGATG CAATAACGAC TGCAACCTCG GTGTCCATTC TTAATTCTTT GTTTATGGTT TTGTACGATT CCATGTAAAA GAAGGATAGA ATTTGAACTG
```

Cut

Supplemental Table 4. PCR confirmation of founder line with expected deletion.

Sample	Exdel	Seq candidate
CR1646-1	NO	
CR1646-2	NO	
CR1646-3	NO	
CR1646-4	YES	
CR1646-5	NO	
CR1646-6	YES	YES
CR1646-7	NO	
CR1646-8	YES	
CR1646-9	NO	

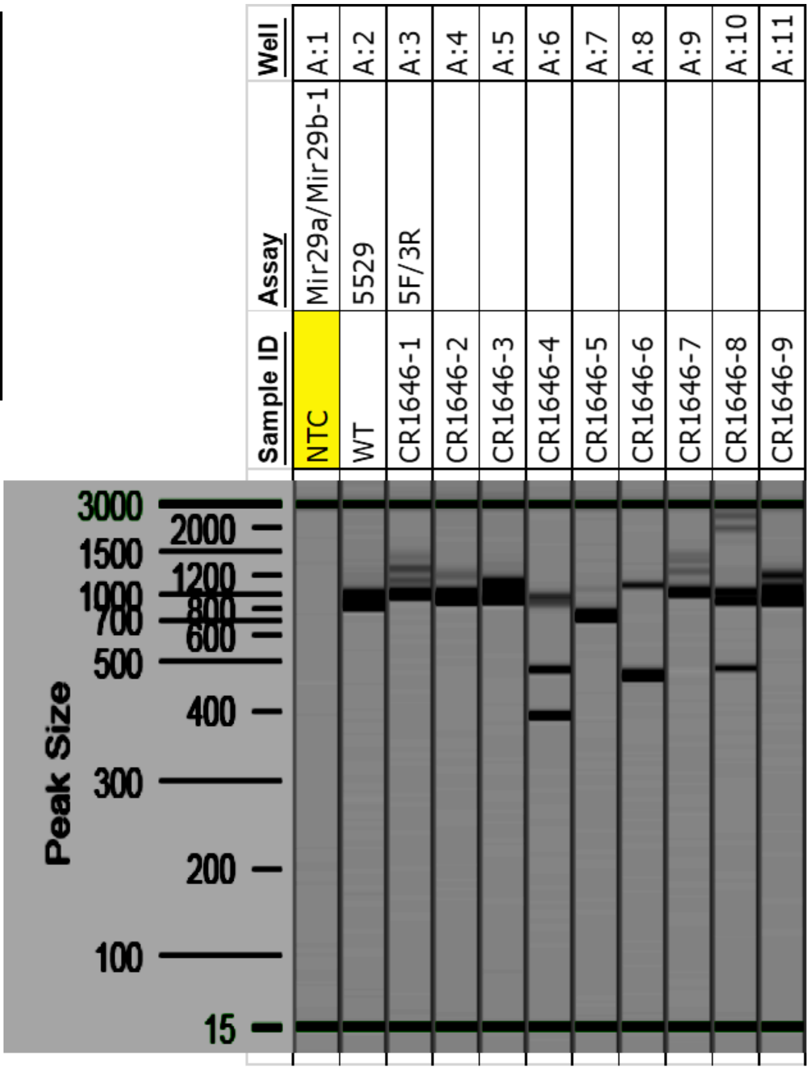

Supplemental Table 5. Sequence confirmation of founder line with expected homologous recombination.

Forward:

TGCNCGTGAATGTGTAAGCCTCGTGCTCACTGTCCCACGTCTGCTCTCGGGTGAGGGCTCAGTTACCATTTGGAGGGAAGAATGCCATGAGTGACCGACATGTCACA
CCCCCACCCCCACCTGCTAGATGAGGTCAGCATAGGATCGCCTGAAATTAGGAACTATTGCACGGACTTCACCTTCCCTCTATAACTTCGTATAGCATACATTATACGA
AGTTATGAATTCCCGTAGGAAGGCCACAAAAACAGACGACAAAGCTTCTTCAGGAAGCTGGTTTCATATGGTGGTTTAGATTTAAATAGTGATTGTCTAGCACCATT
TGAAATCAGTGTTCTTGGTGGAGAACTACTTCGCTGCCGACCGCACAAAGAAATGAGTGTACAGCCCTGAAGTAAGTGTCCAGTGTCTAAAACAGGCTACCAGAGC
CTGCCCTCGCCCACCCTGCTTACCTCTGTGTTATGGATAGCTGATTAGTCAACCACCGTTAGAATTGCATACTTGTTCATGTAATAAGCCTTCTCTGGAAGTGGACTCC
ACCATGCTCGGATGAAGACCTACATATACGACAGATTGAAGGCCTGGGCCTTCCCAGTGCACATGACCTCTTGTGACCCCTTAGAGGATGACTGATTTCTTTTGNTGT
TNNNANNNAANANNNNTNTCTAGCACCATCTGAAATCGGTTANNATGANTGNTGACA

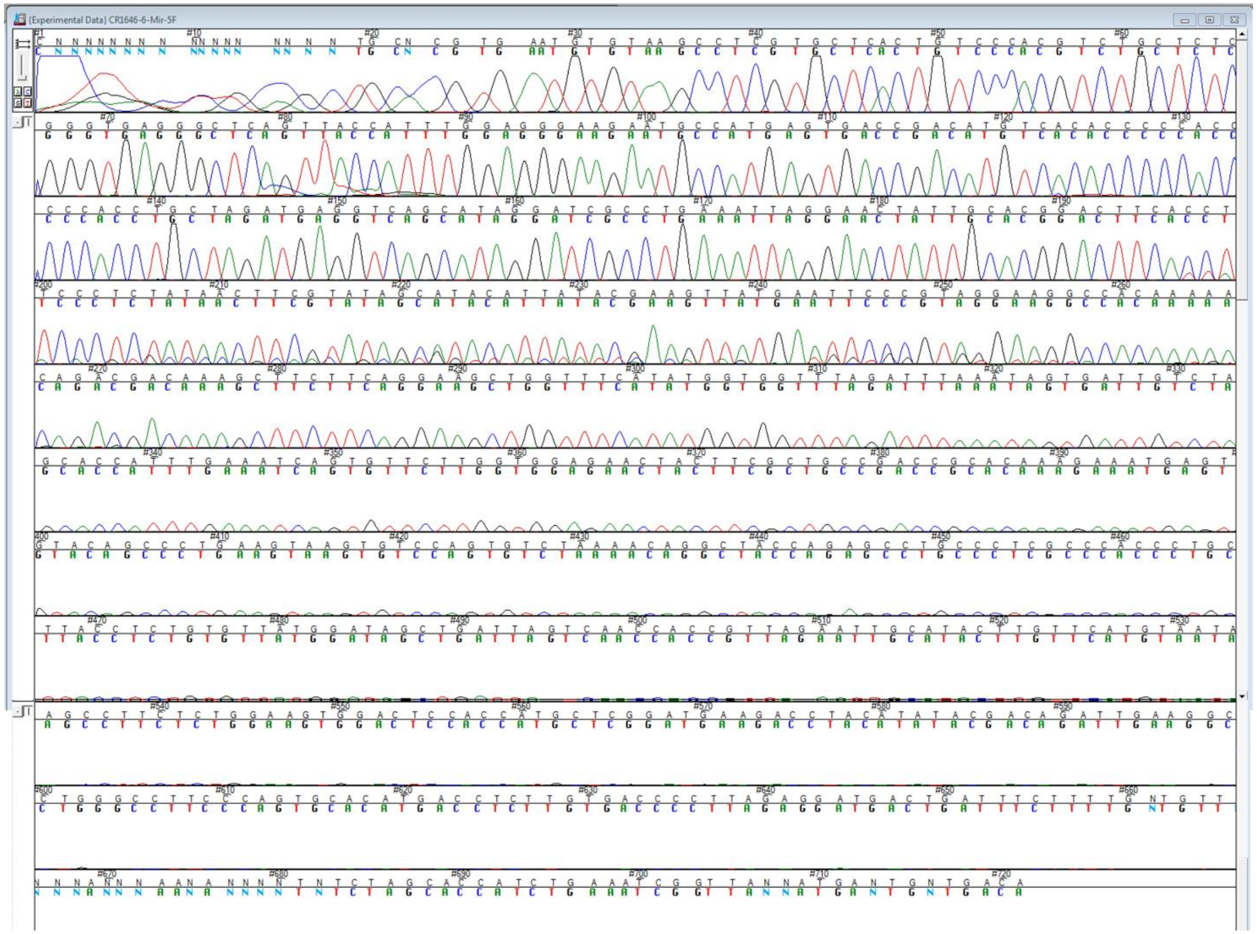

Reverse:

NGGCAGGCTAGGTGACTATCCCTATGTGAGCTGTTAGAACCTTATAGAAGTAAATATCCCCTGCTCCTCTCACATTGACTGGTCAGTCATTGGTTTGGCCCTTTATCCT
CTAGTTACCGTCAAATCTGCAACCCATACACACTTTCTAAAACACACCCACCATCACTATGTGAATAGTTAACCATGCTGTTGCTGGGGCACGTGTTAATGAAAGTCA
AGTTTAAGATAGGAAGAAAATGTACCTTAGGAATTCATAACTTCGTATAATGTATGCTATACGAAGTTATCATGTTTTGGTATTTGTTTCTTAATTCTTACCTGTGGCT
CCAACGTCAGCAATAACGTAGTCAGCTGAACGGTGCTCTTCCCCAATCATTATAACCGATTTCAGATGGTGCTAGAAAATTCTATTGACTCTGAACACCAAAAGAAAT
CAGTCATCCTCTAAGGGGTCACAAGAGGTCATGTGCACTGGGAAGGCCCAGGCCTTCAATCTGTCGTATATGTAGGTCTTCATCCGAGCATGGTGGAGTCCACTTCC
AGAGAAGGCTTATTACATGAACAAGTATGCAATTCTAACGGTGGTTGACTAATCAGCTATCCATAACACAGAGGTAAGCNGGGTGGGCGAGGGCAGGCTCTGGTA
GCCTGTTTTAGACACTGGACACTTACTTCAGGGCTGTACACTCATTTCTTTGTGCGGTCGGCAGCGAANTAGTTCTCCACCAAGAACACTGATTTCAAATGGTGCTAG
ACATCACTATTANTCTAAACCACCATATGAAACCANNTCCTGNNAAGCTNTGTCGACTGTTNNGTCG

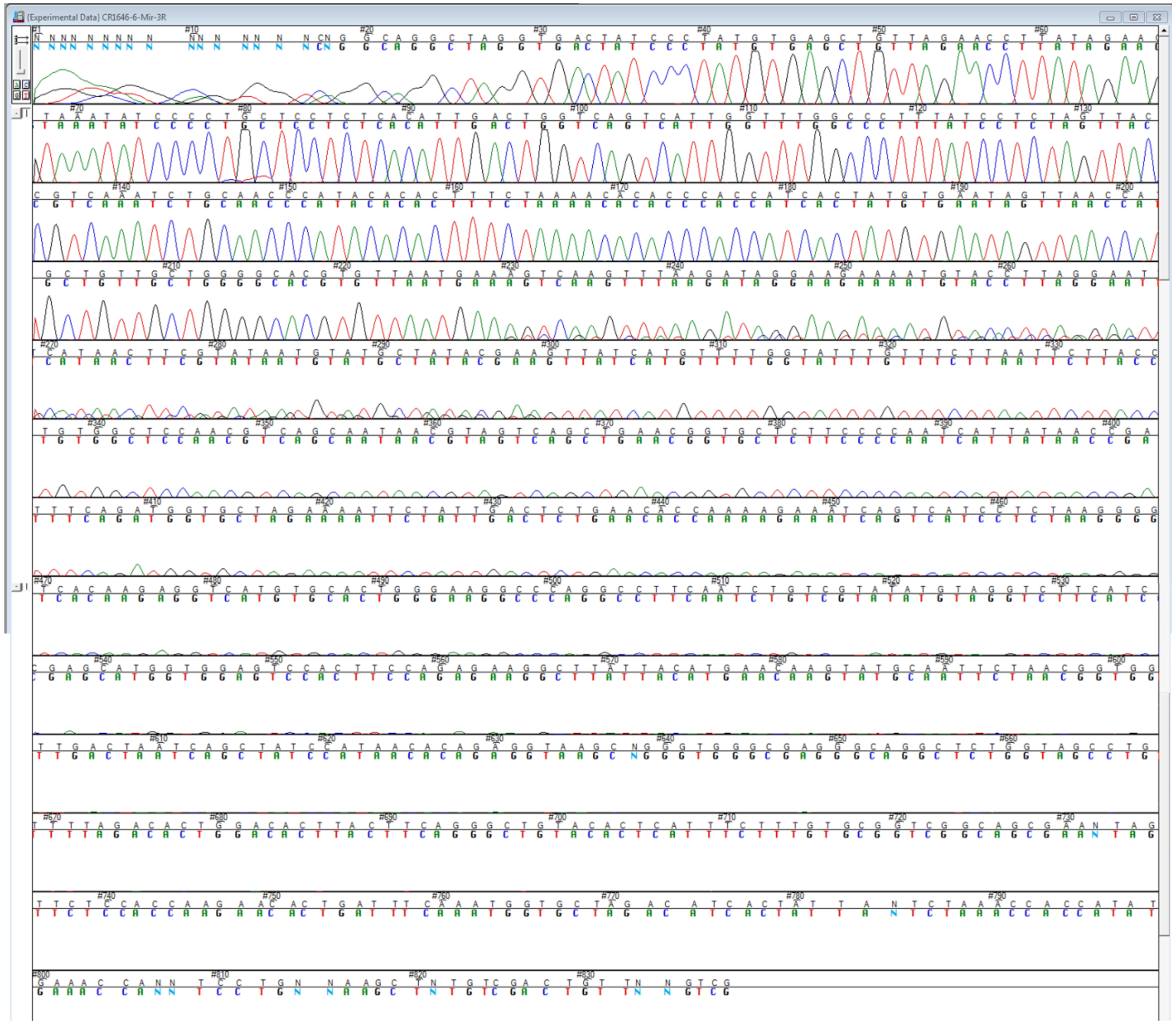

CR1649-8

Forward:

CNGCACCGTGATGTGTAAGCCTCGTGCTCACTGTCCCACGTCTGCTCTCGGGTGAGGGCTCAGTTACCATTTGGAGGGAAGAATGCCATGAGTGACCGACATGTCAC
ACCCCCACCCCCACCTGCTAGATGAGGTCAGCATAGGATCGCCTGAAATTAGGAACTATTGCACGGACTTCATTAACACGTGCCCCAGCAACAGCATGGTTAACTATT
CACATAGTGATGGTGGGTGTGTTTTAGAAAGTGTG

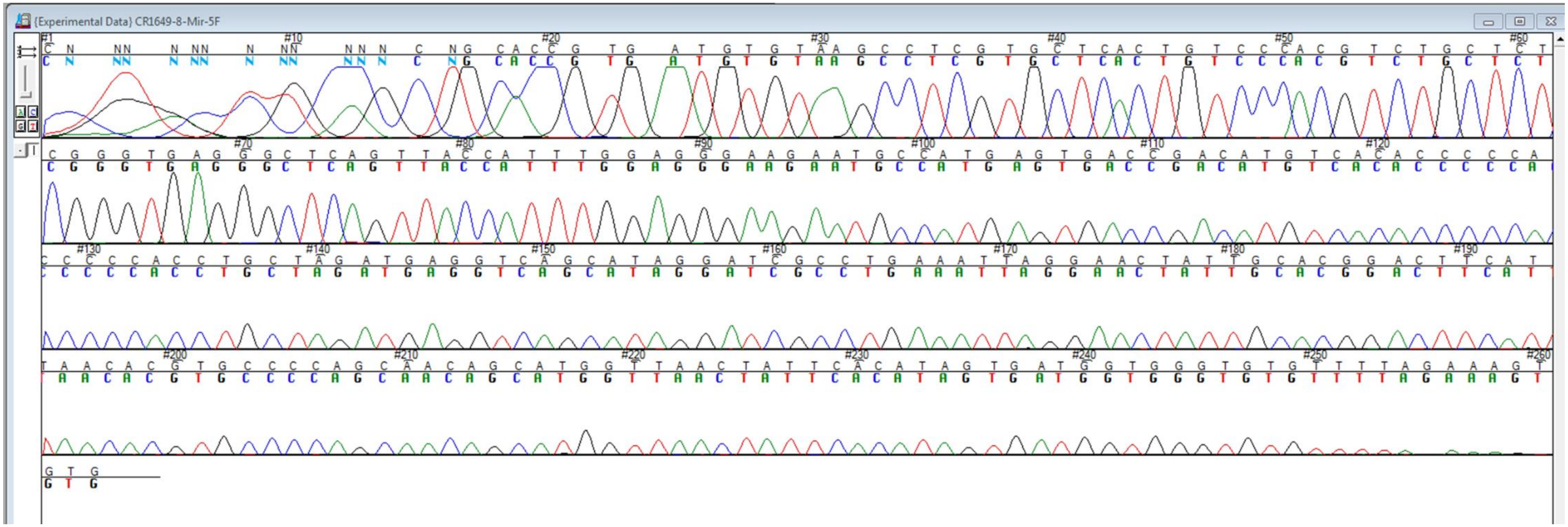

Reverse:

GTCAGGCAGGCTAGGTGACTATCCCTATGTGAGCTGTTAGAACCTTATAGAAGTAAATATCCCCTGCTCCTCTCACATTGACTGGTCAGTCATTGGTTTGGCCCTTTAT
CCTCTAGTTACCGTCAAATCTGCAACCCATACACACTTTCTAAAACACACCCACCATCACTATGTGAATAGTTAACCATGCTGTTGCTGGGGCACGTGTTAATGAAGTC
CGTGCAATAGTTCCTAATTTCAGGCGATCCTATGCTGACCTCATCTAGCAGGTGGGGGTGGGGGTG

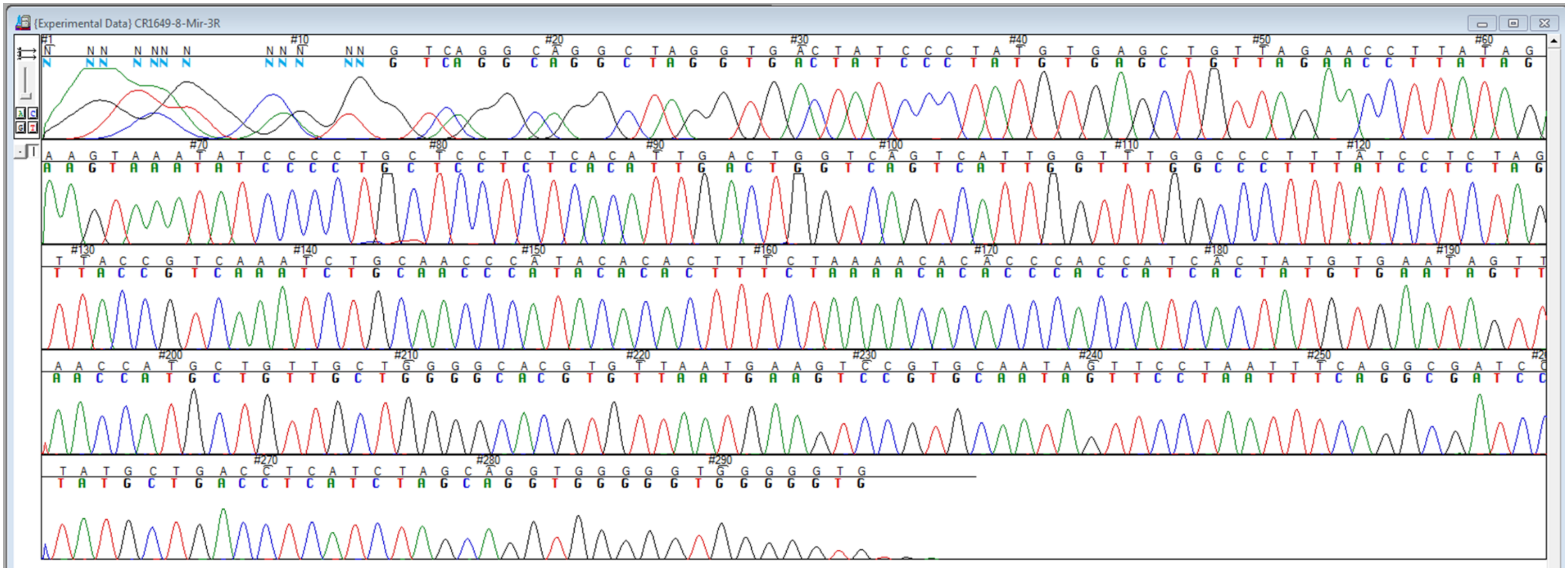

CR1646-6 FWD (5’):

```
Query  1    TGAGCTCTGCCACGCTGCACCGTGAATGTGTAAGCCTCGTGCTCACTGTCCCACGTCTGC  60
            ||        | ||        ||||||||||||||||||||||||||||||||||||||
Sbjct  1    TG--------CNCG--------TGAATGTGTAAGCCTCGTGCTCACTGTCCCACGTCTGC  44

Query  61   TCTCGGGTGAGGGCTCAGTTACCATTTGGAGGGAAGAATGCCATGAGTGACCGACATGTC  120
            ||||||||||||||||||||||||||||||||||||||||||||||||||||||||||||
Sbjct  45   TCTCGGGTGAGGGCTCAGTTACCATTTGGAGGGAAGAATGCCATGAGTGACCGACATGTC  104

Query  121  ACACCCCCACCCCCACCTGCTAGATGAGGTCAGCATAGGATCGCCTGAAATTAGGAACTA  180
            ||||||||||||||||||||||||||||||||||||||||||||||||||||||||||||
Sbjct  105  ACACCCCCACCCCCACCTGCTAGATGAGGTCAGCATAGGATCGCCTGAAATTAGGAACTA  164

Query  181  TTGCACGGACTTCACCTTCCCTCTATAACTTCGTATAGCATACATTATACGAAGTTATGA  240
            ||||||||||||||||||||||||||||||||||||||||||||||||||||||||||||
Sbjct  165  TTGCACGGACTTCACCTTCCCTCTATAACTTCGTATAGCATACATTATACGAAGTTATGA  224

Query  241  ATTCCCGTAGGAAGGCCACAAAAACAGACGACAAAGCTTCTTCAGGAAGCTGGTTTCATA  300
            ||||||||||||||||||||||||||||||||||||||||||||||||||||||||||||
Sbjct  225  ATTCCCGTAGGAAGGCCACAAAAACAGACGACAAAGCTTCTTCAGGAAGCTGGTTTCATA  284

Query  301  TGGTGGTTTAGATTTAAATAGTGATTGTCTAGCACCATTTGAAATCAGTGTTCTTGGTGG  360
            ||||||||||||||||||||||||||||||||||||||||||||||||||||||||||||
Sbjct  285  TGGTGGTTTAGATTTAAATAGTGATTGTCTAGCACCATTTGAAATCAGTGTTCTTGGTGG  344

Query  361  AGAACTACTTCGCTGCCGACCGCACAAAGAAATGAGTGTACAGCCCTGAAGTAAGTGTCC  420
            ||||||||||||||||||||||||||||||||||||||||||||||||||||||||||||
Sbjct  345  AGAACTACTTCGCTGCCGACCGCACAAAGAAATGAGTGTACAGCCCTGAAGTAAGTGTCC  404

Query  421  AGTGTCTAAAACAGGCTACCAGAGCCTGCCCTCGCCCACCCTGCTTACCTCTGTGTTATG  480
            ||||||||||||||||||||||||||||||||||||||||||||||||||||||||||||
Sbjct  405  AGTGTCTAAAACAGGCTACCAGAGCCTGCCCTCGCCCACCCTGCTTACCTCTGTGTTATG  464

Query  481  GATAGCTGATTAGTCAACCACCGTTAGAATTGCATACTTGTTCATGTAATAAGCCTTCTC  540
            ||||||||||||||||||||||||||||||||||||||||||||||||||||||||||||
Sbjct  465  GATAGCTGATTAGTCAACCACCGTTAGAATTGCATACTTGTTCATGTAATAAGCCTTCTC  524

Query  541  TGGAAGTGGACTCCACCATGCTCGGATGAAGACCTACATATACGACAGATTGAAGGCCTG  600
            ||||||||||||||||||||||||||||||||||||||||||||||||||||||||||||
Sbjct  525  TGGAAGTGGACTCCACCATGCTCGGATGAAGACCTACATATACGACAGATTGAAGGCCTG  584

Query  601  GGCCTTCCCAGTGCACATGACCTCTTGTGACCCCTTAGAGGATGACTGATTTCTTTTGGT  660
            |||||||||||||||||||||||||||||||||||||||||||||||||||||||||| |
Sbjct  585  GGCCTTCCCAGTGCACATGACCTCTTGTGACCCCTTAGAGGATGACTGATTTCTTTTGNT  644
```

CR1646-6 REV (3'):

```
Query  1    CAGGCAGGCTAGGTGACTATCCCTATGTGAGCTGTTAGAACCTTATAGAAGTAAATATCC  60
              ||||||||||||||||||||||||||||||||||||||||||||||||||||||||||
Sbjct  1    N-GGCAGGCTAGGTGACTATCCCTATGTGAGCTGTTAGAACCTTATAGAAGTAAATATCC  59

Query  61   CCTGCTCCTCTCACATTGACTGGTCAGTCATTGGTTTGGCCCTTTATCCTCTAGTTACCG  120
            ||||||||||||||||||||||||||||||||||||||||||||||||||||||||||||
Sbjct  60   CCTGCTCCTCTCACATTGACTGGTCAGTCATTGGTTTGGCCCTTTATCCTCTAGTTACCG  119

Query  121  TCAAATCTGCAACCCATACACACTTTCTAAAACACACCCACCATCACTATGTGAATAGTT  180
            ||||||||||||||||||||||||||||||||||||||||||||||||||||||||||||
Sbjct  120  TCAAATCTGCAACCCATACACACTTTCTAAAACACACCCACCATCACTATGTGAATAGTT  179

Query  181  AACCATGCTGTTGCTGGGGCACGTGTTAATGAAAGTCAAGTTTAAGATAGGAAGAAAATG  240
            ||||||||||||||||||||||||||||||||||||||||||||||||||||||||||||
Sbjct  180  AACCATGCTGTTGCTGGGGCACGTGTTAATGAAAGTCAAGTTTAAGATAGGAAGAAAATG  239

Query  241  TACCTTAGGAATTCATAACTTCGTATAATGTATGCTATACGAAGTTATCATGTTTTGGTA  300
            ||||||||||||||||||||||||||||||||||||||||||||||||||||||||||||
Sbjct  240  TACCTTAGGAATTCATAACTTCGTATAATGTATGCTATACGAAGTTATCATGTTTTGGTA  299

Query  301  TTTGTTTCTTAATTCTTACCTGTGGCTCCAACGTCAGCAATAACGTAGTCAGCTGAACGG  360
            ||||||||||||||||||||||||||||||||||||||||||||||||||||||||||||
Sbjct  300  TTTGTTTCTTAATTCTTACCTGTGGCTCCAACGTCAGCAATAACGTAGTCAGCTGAACGG  359

Query  361  TGCTCTTCCCCAATCATTATAACCGATTTCAGATGGTGCTAGAAAATTCTATTGACTCTG  420
            ||||||||||||||||||||||||||||||||||||||||||||||||||||||||||||
Sbjct  360  TGCTCTTCCCCAATCATTATAACCGATTTCAGATGGTGCTAGAAAATTCTATTGACTCTG  419

Query  421  AACACCAAAAGAAATCAGTCATCCTCTAAGGGGTCACAAGAGGTCATGTGCACTGGGAAG  480
            ||||||||||||||||||||||||||||||||||||||||||||||||||||||||||||
Sbjct  420  AACACCAAAAGAAATCAGTCATCCTCTAAGGGGTCACAAGAGGTCATGTGCACTGGGAAG  479

Query  481  GCCCAGGCCTTCAATCTGTCGTATATGTAGGTCTTCATCCGAGCATGGTGGAGTCCACTT  540
            ||||||||||||||||||||||||||||||||||||||||||||||||||||||||||||
Sbjct  480  GCCCAGGCCTTCAATCTGTCGTATATGTAGGTCTTCATCCGAGCATGGTGGAGTCCACTT  539

Query  541  CCAGAGAAGGCTTATTACATGAACAAGTATGCAATTCTAACGGTGGTTGACTAATCAGCT  600
            ||||||||||||||||||||||||||||||||||||||||||||||||||||||||||||
Sbjct  540  CCAGAGAAGGCTTATTACATGAACAAGTATGCAATTCTAACGGTGGTTGACTAATCAGCT  599

Query  601  ATCCATAACACAGAGGTAAGCAGGGTGGGCGAGGGCAGGCTCTGGTAGCCTGTTTTAGAC  660
            ||||||||||||||||||||| ||||||||||||||||||||||||||||||||||||||
Sbjct  600  ATCCATAACACAGAGGTAAGCNGGGTGGGCGAGGGCAGGCTCTGGTAGCCTGTTTTAGAC  659

Query  661  ACTGGACACTTACTTCAGGGCTGTACACTCATTTCTTTGTGCGGTCGGCAGCGAAGTAGT  720
            ||||||||||||||||||||||||||||||||||||||||||||||||||||||| ||||
Sbjct  660  ACTGGACACTTACTTCAGGGCTGTACACTCATTTCTTTGTGCGGTCGGCAGCGAANTAGT  719

Query  721  TCTCCACCAAGAACACTGATTTCAAATGGTGCTAGACAATCACTATTTAAATCTAAACCA  780
            |||||||||||||||||||||||||||||||||||||| ||||||||  | |||||||||
Sbjct  720  TCTCCACCAAGAACACTGATTTCAAATGGTGCTAGACA-TCACTATT--ANTCTAAACCA  776
```

CR1649-8 (5’) NOTE: appears to have sequence from the deletion. REPEAT NECESSARY.

```
Query  1    TGAGCTCTGCCACGCTGCACCGTGAATGTGTAAGCCTCGTGCTCACTGTCCCACGTCTGC  60
                          | ||||||||| ||||||||||||||||||||||||||||||||||
Sbjct  1                  CNGCACCGTGA-TGTGTAAGCCTCGTGCTCACTGTCCCACGTCTGC  45

Query  61   TCTCGGGTGAGGGCTCAGTTACCATTTGGAGGGAAGAATGCCATGAGTGACCGACATGTC  120
            ||||||||||||||||||||||||||||||||||||||||||||||||||||||||||||
Sbjct  46   TCTCGGGTGAGGGCTCAGTTACCATTTGGAGGGAAGAATGCCATGAGTGACCGACATGTC  105

Query  121  ACACCCCCACCCCCACCTGCTAGATGAGGTCAGCATAGGATCGCCTGAAATTAGGAACTA  180
            ||||||||||||||||||||||||||||||||||||||||||||||||||||||||||||
Sbjct  106  ACACCCCCACCCCCACCTGCTAGATGAGGTCAGCATAGGATCGCCTGAAATTAGGAACTA  165

Query  181  TTGCACGGACTTCACCTTCCCTCTATAACTTCGTATAGCATACATTATACGAAGTTATGA  240
            ||||||||||||||
Sbjct  166  TTGCACGGACTTCA----------------------------------------------  179

Query  241  ATTCCCGTAGGAAGGCCACAAAAACAGACGACAAAGCTTCTTCAGGAAGCTGGTTTCATA  300

Sbjct       ------------------------------------------------------------

Query  301  TGGTGGTTTAGATTTAAATAGTGATTGTCTAGCACCATTTGAAATCAGTGTTCTTGGTGG  360

Sbjct       ------------------------------------------------------------

Query  361  AGAACTACTTCGCTGCCGACCGCACAAAGAAATGAGTGTACAGCCCTGAAGTAAGTGTCC  420

Sbjct       ------------------------------------------------------------

Query  421  AGTGTCTAAAACAGGCTACCAGAGCCTGCCCTCGCCCACCCTGCTTACCTCTGTGTTATG  480

Sbjct       ------------------------------------------------------------

Query  481  GATAGCTGATTAGTCAACCACCGTTAGAATTGCATACTTGTTCATGTAATAAGCCTTCTC  540

Sbjct       ------------------------------------------------------------

Query  541  TGGAAGTGGACTCCACCATGCTCGGATGAAGACCTACATATACGACAGATTGAAGGCCTG  600

Sbjct       ------------------------------------------------------------

Query  601  GGCCTTCCCAGTGCACATGACCTCTTGTGACCCCTTAGAGGATGACTGATTTCTTTTGGT  660

Sbjct       ------------------------------------------------------------

Query  661  GTTCAGAGTCAATAGAATTTTCTAGCACCATCTGAAATCGGTTATAATGATTGGGGAAGA  720

Sbjct       ------------------------------------------------------------

Query  721  GCACCGTTCAGCTGACTACGTTATTGCTGACGTTGGAGCCACAGGTAAGAATTAAGAAAC  780

Sbjct       ------------------------------------------------------------

Query  781  AAATACCAAAACATGATAACTTCGTATAGCATACATTATACGAAGTTATGAATTCCTAAG  840

Sbjct       ------------------------------------------------------------

Query  841  GTACATTTTCTTCCTATCTTAAACTTGACTTTCATTAACACGTGCCCCAGCAACAGCATG  900
                                              ||||||||||||||||||||||||||
Sbjct  180  ----------------------------------TTAACACGTGCCCCAGCAACAGCATG  205

Query  901  GTTAACTATTCACATAGTGATGGTGGGTGTGTTTTAGAAAGTGTGTATGGGTTGCAGATT  960
            |||||||||||||||||||||||||||||||||||||||||||||
Sbjct  206  GTTAACTATTCACATAGTGATGGTGGGTGTGTTTTAGAAAGTGTG                250
```

CR1649-8 (3') NOTE: appears to have sequence from the deletion. REPEAT NECESSARY.

```
Query  1     ATGCAATGGCCAGTGGTCAGGCAGGCTAGGTGACTATCCCTATGTGAGCTGTTAGAACCT  60
                            ||||||||||||||||||||||||||||||||||||||||||||||
Sbjct  1                    GTCAGGCAGGCTAGGTGACTATCCCTATGTGAGCTGTTAGAACCT  45

Query  61    TATAGAAGTAAATATCCCCTGCTCCTCTCACATTGACTGGTCAGTCATTGGTTTGGCCCT  120
             ||||||||||||||||||||||||||||||||||||||||||||||||||||||||||||
Sbjct  46    TATAGAAGTAAATATCCCCTGCTCCTCTCACATTGACTGGTCAGTCATTGGTTTGGCCCT  105

Query  121   TTATCCTCTAGTTACCGTCAAATCTGCAACCCATACACACTTTCTAAAACACACCCACCA  180
             ||||||||||||||||||||||||||||||||||||||||||||||||||||||||||||
Sbjct  106   TTATCCTCTAGTTACCGTCAAATCTGCAACCCATACACACTTTCTAAAACACACCCACCA  165

Query  181   TCACTATGTGAATAGTTAACCATGCTGTTGCTGGGGCACGTGTTAATGAAAGTCAAGTTT  240
             ||||||||||||||||||||||||||||||||||||||||||||||||||
Sbjct  166   TCACTATGTGAATAGTTAACCATGCTGTTGCTGGGGCACGTGTTAATGAA----------  215

Query  241   AAGATAGGAAGAAAATGTACCTTAGGAATTCATAACTTCGTATAATGTATGCTATACGAA  300

Sbjct        ------------------------------------------------------------

Query  301   GTTATCATGTTTTGGTATTTGTTTCTTAATTCTTACCTGTGGCTCCAACGTCAGCAATAA  360

Sbjct        ------------------------------------------------------------

Query  361   CGTAGTCAGCTGAACGGTGCTCTTCCCCAATCATTATAACCGATTTCAGATGGTGCTAGA  420

Sbjct        ------------------------------------------------------------

Query  421   AAATTCTATTGACTCTGAACACCAAAAGAAATCAGTCATCCTCTAAGGGGTCACAAGAGG  480

Sbjct        ------------------------------------------------------------

Query  481   TCATGTGCACTGGGAAGGCCCAGGCCTTCAATCTGTCGTATATGTAGGTCTTCATCCGAG  540

Sbjct        ------------------------------------------------------------

Query  541   CATGGTGGAGTCCACTTCCAGAGAAGGCTTATTACATGAACAAGTATGCAATTCTAACGG  600

Sbjct        ------------------------------------------------------------

Query  601   TGGTTGACTAATCAGCTATCCATAACACAGAGGTAAGCAGGGTGGGCGAGGGCAGGCTCT  660

Sbjct        ------------------------------------------------------------

Query  661   GGTAGCCTGTTTTAGACACTGGACACTTACTTCAGGGCTGTACACTCATTTCTTTGTGCG  720

Sbjct        ------------------------------------------------------------

Query  721   GTCGGCAGCGAAGTAGTTCTCCACCAAGAACACTGATTTCAAATGGTGCTAGACAATCAC  780

Sbjct        ------------------------------------------------------------

Query  781   TATTTAAATCTAAACCACCATATGAAACCAGCTTCCTGAAGAAGCTTTGTCGTCTGTTTT  840

Sbjct        ------------------------------------------------------------

Query  841   TGTGGCCTTCCTACGGGAATTCATAACTTCGTATAATGTATGCTATACGAAGTTATAGAG  900

Sbjct        ------------------------------------------------------------

Query  901   GGAAGGTGAAGTCCGTGCAATAGTTCCTAATTTCAGGCGATCCTATGCTGACCTCATCTA  960
                       ||||||||||||||||||||||||||||||||||||||||||||||||||
Sbjct  216   ----------GTCCGTGCAATAGTTCCTAATTTCAGGCGATCCTATGCTGACCTCATCTA  265

Query  961   GCAGGTGGGGGTGGGGGTGTGACATGTCGGTCACTCATGGCATTCTTCCCTCCAAATGGT  1020
             |||||||||||||||||||
Sbjct  266   GCAGGTGGGGGTGGGGGTG                                           284
```
